# Developing diazirine-based chemical probes to identify histone modification ‘readers’ and ‘erasers’†
†Electronic supplementary information (ESI) available: experimental methods and figures. See DOI: 10.1039/c4sc02328e



**DOI:** 10.1039/c4sc02328e

**Published:** 2014-11-12

**Authors:** Tangpo Yang, Zheng Liu, Xiang David Li

**Affiliations:** a Department of Chemistry , The University of Hong Kong , Pokfulam Road , Hong Kong , China . Email: xiangli@hku.hk

## Abstract

Post translational modifications (PTMs, *e.g.*, phosphorylation, acetylation and methylation) of histone play important roles in regulating many fundamental cellular processes such as gene transcription, DNA replication and damage repair. While ‘writer’ and ‘eraser’ enzymes modify histones by catalyzing the addition and removal of histone PTMs, ‘reader’ proteins recognize these modified histones and ‘translate’ the PTMs by executing distinct cellular programs. Therefore, identification of the regulating enzymes and binding partners of histone PTMs is essential for understanding their regulatory mechanisms and cellular functions. Here we report the development of diazirine-based photoaffinity probes for identification of ‘readers’ and ‘erasers’ of histone PTMs. When compared with previously described benzophenone-based photoaffinity probes, the present probes demonstrate significantly improved photo-cross-linking rates, yields and specificities for capturing proteins that recognize a trimethylation mark on histone H3 lysine 4 (H3K4Me_3_). Furthermore, we show that the diazirine-based probes can also be used to identify enzymes that catalyse the removal of histone lysine acetylation and malonylation. This study provides new chemical tools for examining PTM-mediated protein–protein interactions and broadens the scope of our photo-cross-linking strategy from finding histone PTM ‘readers’ to identifying dynamic and transient interactions between PTMs and their ‘erasers’.

## Abstract 2

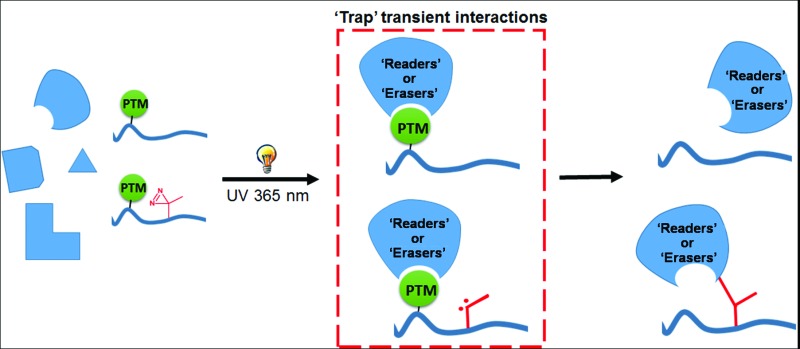
New chemical tools to ‘trap’ post translational modification (PTM)-mediated protein–protein interactions.

## Introduction

In the nuclei of all eukaryotic cells, genomic DNA is highly compacted into chromatin by wrapping around ‘spools’, which are comprised of the four core histones, H3, H4, H2A and H2B.^1^ The core histones, particularly their unstructured N-terminal ‘tails’, can be covalently modified by enzymes that catalyse the addition and removal of diverse post translational modifications (PTMs), such as acetylation, phosphorylation and methylation.^2,3^ These histone PTMs can directly affect higher-order chromatin structures and can serve as signalling platforms that orchestrate the recruitment of ‘reader’ or ‘effector’ proteins to carry out downstream cellular events.^4,5^ As such, histone PTMs contribute to many fundamental biological processes such as gene transcription, DNA replication and damage repair.^2,6^

To link a particular histone PTM to its downstream biological functions requires comprehensive identification of the ‘readers’ of the PTM. However, it is challenging to find PTM-mediated protein–protein interactions, as PTMs are often present on a small fraction of proteins, with dynamic, or mediating transient and weak interactions. To address this difficulty, a chemical approach has been developed to ‘trap’ proteins that recognize histone PTMs.^7^ This approach relies on the use of histone peptide-based photoaffinity probes that carry (i) PTMs of interest at a stoichiometric level, (ii) a benzophenone-based photoreactive crosslinking group to capture PTM ‘readers’ by converting non-covalent protein–protein interactions into irreversible covalent linkages, and (iii) a terminal alkyne group to enable bioorthorgonal conjugation of fluorescence or affinity tags for the detection or purification of captured proteins. These photoaffinity probes, in combination with stable isotope labelling amino acid in cell culture (SILAC)-based quantitative proteomics technology, have been used to profile the ‘readers’ of histone H3 trimethylation at lysine 4 (H3K4Me_3_) and lysine 9 (H3K9Me_3_) and phosphorylation at threonine 3 (H3T3-Phos).^8,9^ However, this chemical proteomics approach has suffered from low protein labelling efficiency and specificity. For example, in a model study, only less than 5% of total cellular inhibitor of growth protein 2 (ING2), a known histone H3K4Me_3_ ‘reader’, was labelled by an H3K4Me_3_ peptide-based photoaffinity probe (probe **1**),^7^ and the quantitative proteomics analysis revealed that 98% of proteins captured by probe **1** were not specific binders of the histone H3K4Me_3_ mark.^9^ All of these problems are likely caused by the low efficiencies and specificities of the photo-cross-linking reactions mediated by the benzophenone moiety in probe **1**.

It is known that benzophenone requires a long period of irradiation and that its excitation is reversible, which may lead to non-specific labelling.^10^ Besides, steric hindrance of bulky benzophenone is likely to disrupt the protein–probe interactions when it is incorporated too close to the binding sites. In addition to benzophenone, arylazide and diazirine are another two commonly used photoreactive groups. However, the application of arylazide as a photo-cross-linker is limited as it tends to photoisomerize to less reactive dehydroazepine, which leads to low cross-linking yields and specificities.^11^ In addition, arylazide also has a relatively large size that may cause steric hindrance. Diazirine, on the other hand, exhibits more advantages for photoaffinity labelling, including its small size, its short lifetime upon UV irradiation and high subsequent reactivity.^12–16^ Taking these aspects into consideration, here we use diazirine as a photoreactive group to develop new photoaffinity probes for the identification of histone PTM-mediated protein–protein interactions. We directly compare the benzophenone- and diazirine-based probes for their photo-cross-linking rates, yields and specificities in complex protein mixtures and for their steric effects on PTM-mediated protein–protein interactions. We show that the diazirine-based probes have significantly improved performances for capturing histone H3K4Me_3_ ‘readers’. We further demonstrate that our diazirine-based photoaffinity probes can also be used to identify enzymes that catalyse the removal of histone PTMs (*i.e.*, ‘erasers’), including the lysine deacetylases and demalonylase.

## Results and discussion

### Design and synthesis of photoaffinity probes for histone H3K4Me3 ‘readers’

**Fig. 1:**
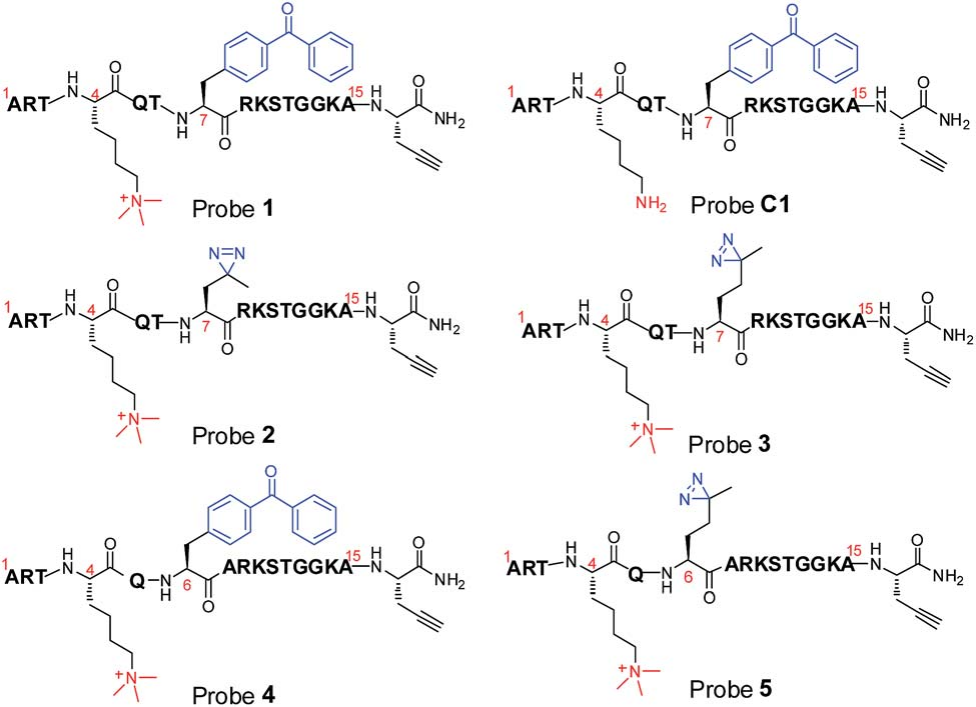
Chemical structures of photoaffinity probes **1–5** and **C1**.

To compare diazirine with benzophenone with respect to their abilities to capture histone PTM ‘readers’, we first focused on the histone H3K4Me_3_ mark as a model system. We designed probes **2** and **3** (Fig. 1) based on a histone H3K4Me_3_ peptide (residues 1–15) in which two diazirine-containing amino acids, photo-leucine (photo-Leu) and photo-methionine (photo-Met), respectively, were used to replace Ala 7, the residue that was replaced by benzoyl phenylalanine (Bpa) in the design of probe **1**. To study the steric effects of the two photoreactive groups on the protein labelling efficiency, probes **4** and **5** (Fig. 1) were designed with the benzophenone and diazirine moieties, respectively, moved one residue closer to the K4-trimethylation site. In addition, unmodified histone H3 probe **C1** (Fig. 1) was designed as a control probe.

**Scheme 1:**
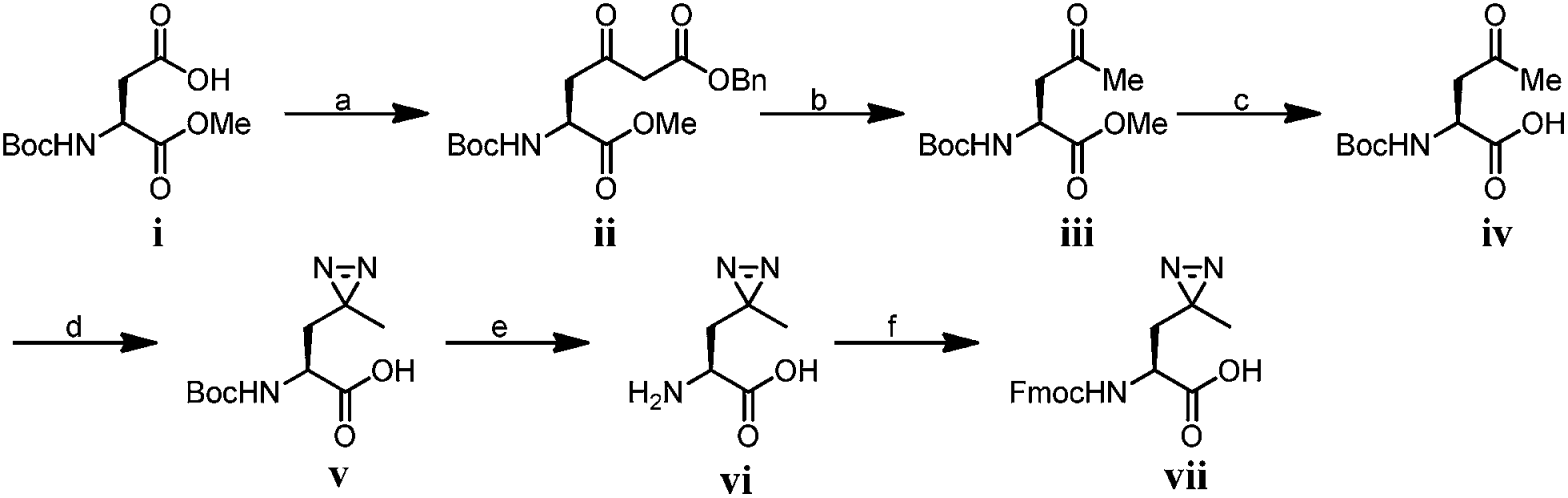
Synthesis of Fmoc-photo-Leu; conditions: (a) 1,1′-carbonyldiimidazole, isopropyl magnesium chloride, monobenzyl malonate, THF, 85%; (b) Pd/C, MeOH, 83%; (c) LiOH, THF/H_2_O (2 : 1), r.t. 98% (d) (1) NH_3_, hydroxylamine-*O*-sulfonic acid; (2) TEA, I_2_, MeOH, 30%; (e) TFA/dichloromethane (1 : 1); (f) Fmoc-OSu, NaHCO_3_, 1,4-dioxane/H_2_O (2 : 1), 97% over two steps.

We first synthesized Fmoc-protected photo-Leu and photo-Met as building blocks for the assembly of the corresponding photoaffinity peptide probes. Fmoc-protected photo-Met was synthesized in five steps following a procedure described in the literature.^17^ The previously reported syntheses of photo-Leu required either an enzymatic resolution step^18^ or use of an expensive unnatural amino acid (4,5-dehydro-leucine) as a starting material.^19^ To avoid these problems, we developed an efficient six-step route to synthesize Fmoc-protected photo-Leu (Scheme 1). Briefly, Boc-protected l-aspartic acid α-methyl ester (**i**) was transformed into the side-chain β-ketoester **ii** by reaction with monobenzyl malonate. Hydrogenolysis of **ii** using Pd/C and H_2_ followed by a simultaneous decarboxylation smoothly gave ketone **iii**,^20^ which was then treated with LiOH to hydrolyze the methyl ester, affording carboxylic acid **iv**. The diazirine moiety was then installed by reaction of **iv** with liquid ammonia followed by hydroxylamine-*O*-sulfonic acid to give the diaziridine intermediate, which was subsequently oxidized to give **v**.^19^ Deprotection of Boc and reprotection with Fmoc-OSu gave compound **vi** and Fmoc-protected photo-Leu (**vii**), respectively. The overall yield of **vii** was 20%. Fmoc-photo-Leu and Fmoc-photo-Met were then used to assemble the diazirine-based peptide probes through solid-phase peptide synthesis (SPPS) with a Fmoc-protection strategy.

### Diazirine-based probes exhibit improved photo-cross-linking efficiencies

We first examined the abilities of benzophenone-containing probe **1** and diazirine-containing probes **2** and **3** to covalently label a known H3K4Me_3_ ‘reader’, spindlin 1 (SPIN1),^9,21^ *in vitro*. The recombinant GST-fused mouse SPIN1 was incubated with probes **1**, **2** and **3**, respectively, and irradiated with UV light for 1 h. The probe-labelled proteins were then conjugated with rhodamine azide (Rho-N_3_) *via* Cu(i)-catalysed azide–alkyne cycloadditions (“click chemistry”). The resulting reaction mixtures were resolved by SDS-PAGE and analysed by in-gel fluorescence scanning. The results showed dose-dependent labelling of SPIN1 by all probes, and the labelling was saturated with around 2 μM of each probe (Fig. S1†).

**Fig. 2:**
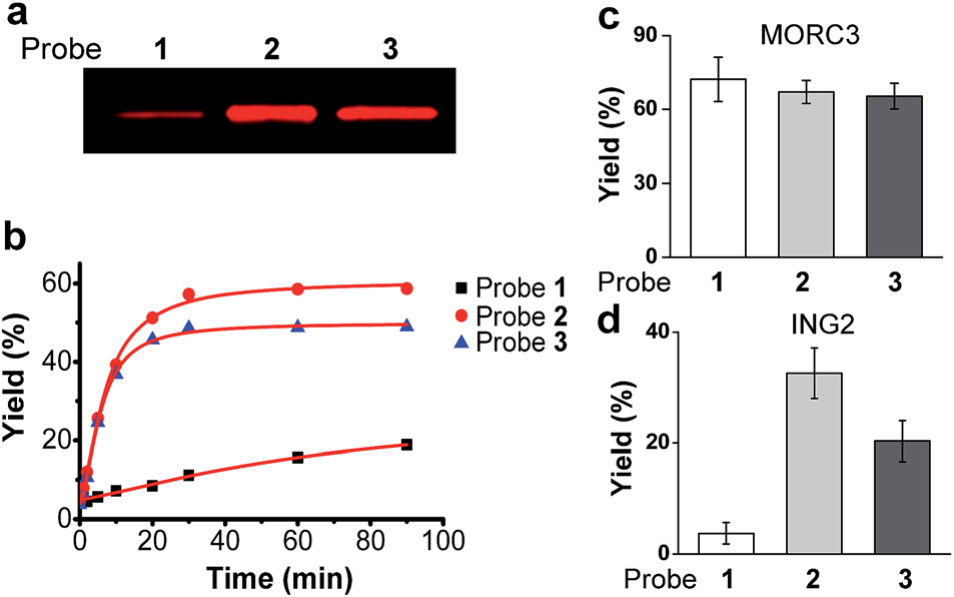
(a) The labelling of SPIN1 by probes **1**, **2** and **3**. Recombinant SPIN1 (20 ng μL^–1^) was incubated with probes **1**, **2**, and **3** (2 μM), respectively. After UV irradiation (365 nm) for 1 h, the labelled SPIN1 was conjugated to a rhodamine-azide tag, resolved by SDS-PAGE, and detected by in-gel fluorescence scanning. (b) Labelling yields of SPIN1 with probes **1**, **2**, and **3** (2 μM) during the course of UV irradiation (365 nm). Labelling yields of (c) MORC3 and (d) ING2 with probes **1**, **2**, and **3** (2 μM), respectively. Data are averages ± s.e. (*n* = 3).

Strikingly, SPIN1 labelled by probe **2** or **3** showed a much stronger fluorescence signal than that labelled by probe **1** (Fig. 2a). To determine the efficiencies of the photo-cross-linking reactions mediated by probes **1–3**, we quantified the amount of protein labelled by each probe during the reaction time. To do that, we first labelled bovine serum albumin (BSA) with an amine-reactive rhodamine dye that has the same fluorophore as Rho-N_3_. After determining the degree of labelling, this rhodamine-labelled BSA (Rho-BSA) was used as a standard to generate a calibration curve for the quantitative analysis of the proteins labelled by the photoaffinity probes (see ESI†). As shown in Fig. S2,† while 16% of SPIN1 was labelled by probe **1** in 1 h, probes **2** and **3** achieved 59% and 49% labelling of the protein, respectively. Given that the three probes should not differ from one another in their click chemistry reactions, this result indicates that diazirine-based probes **2** and **3** have higher photo-cross-linking efficiencies than benzophenone-based probe **1** toward this H3K4Me_3_ ‘reader’.

We next examined the time dependence of the photo-cross-linking reactions mediated by probes **1–3**. As expected, probes **2** and **3** rapidly labelled SPIN1 upon UV irradiation and the labelling yields reached the saturation points (51% for **2** and 46% for **3**) within the first 20 minutes. In contrast, probe **1** labelled only 19% of SPIN1 after 90 minutes irradiation. Inspired by this result, we sought to test whether probes **2** and **3** could also mediate efficient labelling of other histone H3K4Me_3_ ‘readers’. To this end, we chose another two known H3K4Me_3_ binders, ING2^22,23^ and MORC3^9^ that recognize this histone mark with their PHD finger and ZW finger, respectively. Together with SPIN1 that binds H3K4Me_3_ with its double-tudor domain,^24^ these three chosen proteins represent ‘readers’ with three types of distinguished structural domains for H3K4Me_3_ recognition. Each protein was then labelled by probes **1–3** for 20 minutes, respectively, and the labelling yields were determined using the method described above. As shown in Fig. 2d, only 4% of ING2 was captured by probe **1**, whereas probes **2** and **3** labelled the protein with 33% and 20% yields, respectively, indicating the largely improved photo-cross-linking efficiency of these diazirine-based probes. Unlike SPIN1 and ING2, no significant difference in the labelling yields of MORC3 by probes **1–3** was observed (Fig. 2c). Taken together, these data suggest that when compared with the benzophenone-based photoaffinity probe, the diazirine-based probes require much shorter irradiation periods to obtain similar or higher protein labelling yields, which potentially could reduce the possibility of nonspecific labelling and avoid damage to the targeted proteins caused by long exposure times to UV light.

### Diazirine enables robust protein labelling with more flexibility on site of incorporation

**Fig. 3:**
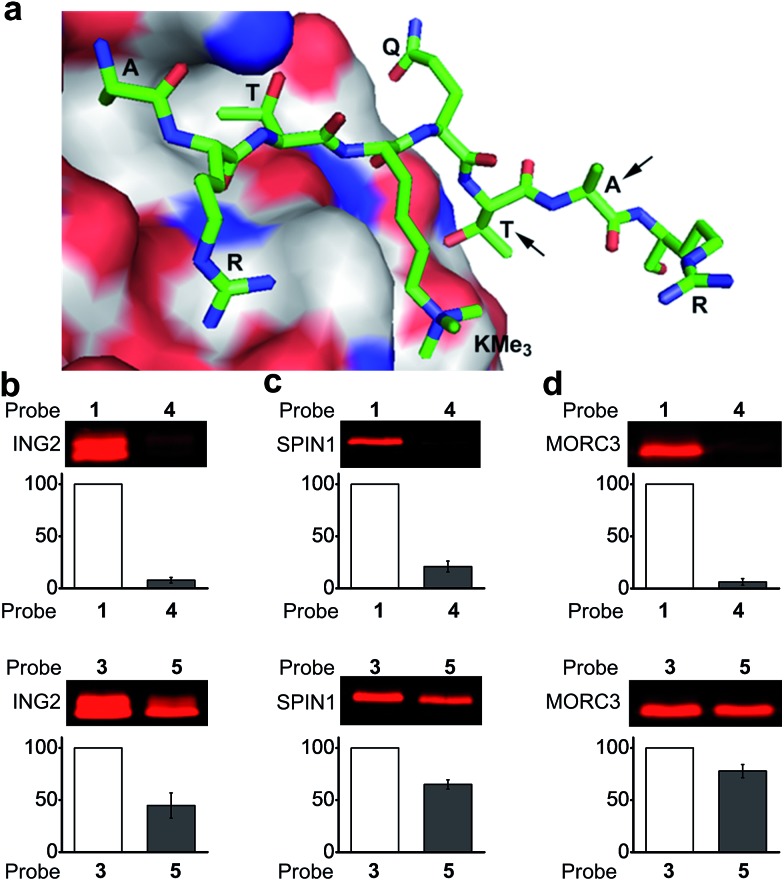
(a) The crystal structure of the PHD finger of ING2 binding to an H3K4Me_3_ peptide (PDB ; 2G6Q). The arrows indicate the residues where the photoreactive groups (*i.e.*, benzophenone and diazirine) were incorporated into probes **1**, **3**, **4** and **5**. The labelling of (b) ING2, (c) SPIN1 and (d) MORC3 by probes **1** and **4** (upper panel), and by probes **3** and **5** (lower panel). The indicated proteins (20 ng μL^–1^) were photo-labelled (365 nm) with probes (2 μM) **1** and **4** for 1 h, and with probes **3** and **5** for 20 minutes, respectively. The labelled proteins were conjugated to rhodamine-azide and detected by in-gel fluorescence scanning. The column charts show the relative fluorescence intensities of the labelled protein bands. The fluorescence intensities of probe **1**- and **3**-labelled proteins were set to 100%. Data are averages ± s.e. (*n* = 2).

To design photoaffinity probes for studying protein–protein interactions, it is important to consider the position in which to incorporate photoreactive groups, which should be close enough to protein binding sites to ensure efficient and specific labelling, but without interfering with protein–protein interactions. We therefore compared the protein labelling efficiencies of benzophenone and diazirine when incorporated into different sites of the peptide probes. The original design of probes **1** and **3** was based on a crystal structure of the PHD finger of ING2 binding to an H3K4Me_3_ peptide.^23^ Since this protein–peptide interaction mainly involves peptide residues from Ala 1 through Thr 6, the photoreactive group, benzophenone, was placed at Ala 7, residue P + 3 relative to the trimethylated Lys 4 (Fig. 3a). To examine the steric effects of benzophenone and diazirine, we incorporated these two photoreactive groups at Thr 6, one residue closer to the modification site, in probes **4** and **5**, respectively. The photo-cross-linking efficiencies of these probes toward ING2 were then examined using the fluorescence-based assay described above.

Unlike probe **1**, probe **4** demonstrated little ability to label ING2. In contrast, probe **5** showed robust labelling of ING2, although with a reduced efficiency when compared with probe **3** (Fig. 3b). In addition to ING2, another two H3K4Me_3_‘readers’, SPIN1 and MORC3, were also robustly labelled by probe **5** but not probe **4** (Fig. 3c and d). These results suggest that due to its bulky structure, benzophenone, when placed too close to the modification site, is likely to disrupt the interaction between the H3K4Me_3_ peptide and its binding partners, whereas the small size of diazirine minimizes this possibility. As a result, although diazirine-based probes **2** and **3** did not show advantages in labelling MORC3 (Fig. 2c), probe **5**, in which the diazirine moiety was moved closer to the modification site, indeed demonstrated a much higher labelling efficiency than the corresponding benzophenone-based probe **4** (Fig. S3†).

### Diazirine-based probes demonstrate less non-specific labelling

**Fig. 4:**
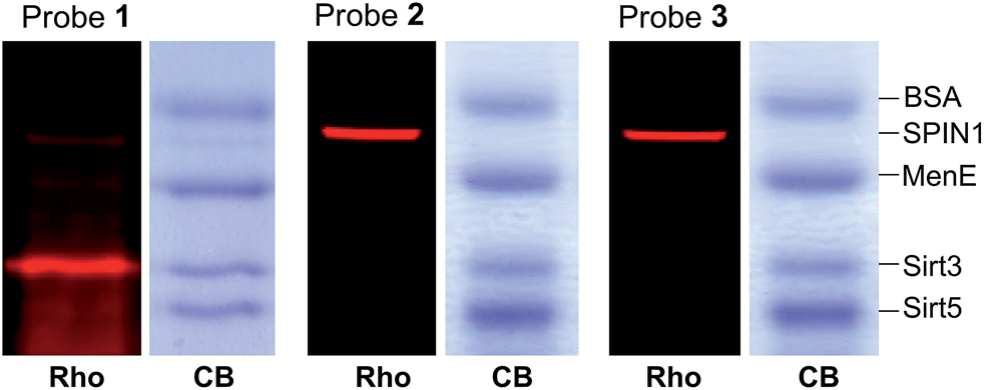
The labelling of a protein mixture of SPIN1 (20 ng μL^–1^), BSA (200 ng μL^–1^), MenE (200 ng μL^–1^), Sirt3 (200 ng μL^–1^) and Sirt5 (200 ng μL^–1^) by probes **1–3** (2 μM), respectively. Rho, rhodamine fluorescence; CB, Coomassie blue.

We next examined the specificity of the photo-cross-linking mediated by the benzophenone- and diazirine-containing probes. We mixed one specific H3K4Me_3_-binding protein (SPIN1) with 10-fold excesses of four other proteins that have not been related to H3K4Me_3_, including BSA, Sirt3, Sirt5 and MenE. Probes **1–3** were then each incubated with this protein mixture. After UV irradiation and click chemistry, the mixtures were resolved by SDS-PAGE and analysed by in-gel fluorescence scanning. As shown in Fig. 4, probes **2** and **3** specifically labelled SPIN1 without interference from the other 4 proteins present in large excesses in the mixtures. However, although able to capture SPIN1, probe **1** showed much stronger labelling of Sirt3, which is not supposed to interact with the histone H3K4Me_3_ peptide.

**Fig. 5:**
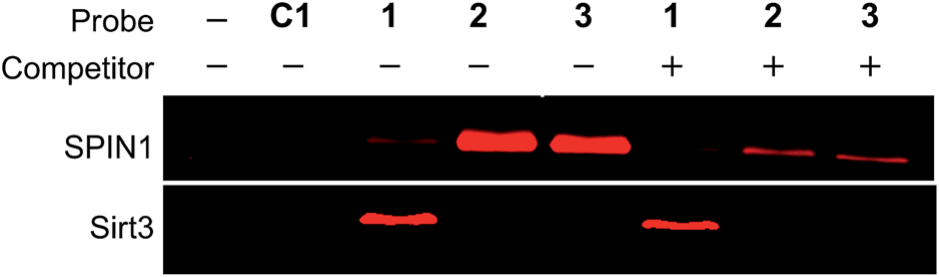
The labelling of SPIN1 (20 ng μL^–1^) by probes **1**, **2** or **3** (2 μM) was inhibited by the H3K4Me_3_ peptide (30 μM), whereas probe **1**-mediated labelling of Sirt3 was not affected by the peptide, indicating that Sirt3 is not a specific binder of H3K4Me_3_.

To rule out the possibility that the labelling of Sirt3 by **1** is not caused by a specific recognition of the histone H3K4Me_3_ mark, we compared the behaviours of SPIN1 and Sirt3 in a series of photo-cross-linking experiments. Interestingly, similarly to SPIN1, Sirt3 was labelled only by probe **1** and not by probe **C1**, an unmodified H3 control probe, indicating that the labelling of Sirt3 is K4-methylation-dependent. However, unlike for SPIN1, the labelling of Sirt3 by probe **1** (2 μM) cannot be competed with by an H3K4Me_3_ peptide (30 μM) (Fig. 5). This result suggests that Sirt3 has no or a very weak interaction with the H3K4Me_3_ peptide, and that the interaction between Sirt3 and probe **1**, which leads to the robust labelling of the protein, might involve both trimethylation at Lys 4 and the hydrophobic benzophenone at Ala 7 of the probe.

**Fig. 6:**
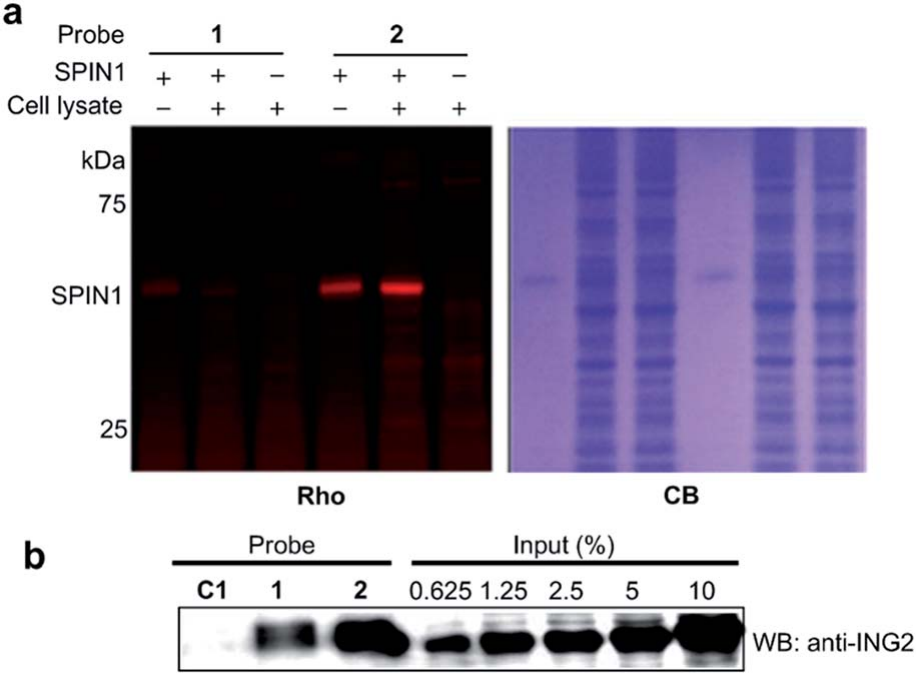
(a) The labelling of SPIN1 (20 ng μL^–1^) from a whole-cell lysate (1.5 mg mL^–1^) by probes **1** and **2** (2 μM). Probe **2** demonstrated a higher efficiency in the selective labelling of SPIN1. (b) Western blot analysis shows the enrichment of endogenous ING2 from the lysates of HeLa S3 cells by probes **C1**, **1** and **2**. The cell lysate (3 mg mL^–1^) was incubated with each probe (2 μM) for 15 min. After UV irradiation (365 nm, 15 min for probes **C1** and **1**, 10 min for probe **2**), the labelled proteins were conjugated to biotin through click chemistry. Following enrichment by streptavidin beads, the enriched proteins were resolved by SDS-PAGE and analyzed by western blotting. Rho, rhodamine fluorescence; CB, Coomassie blue; WB: western blot.

We next analysed the ability of the photoaffinity probes to specifically label H3K4Me_3_ ‘readers’ in complex proteomes. SPIN1 was added to HeLa S3 cell lysates and subjected to photo-cross-linking reactions using probes **1** and **2**. We found that while both the probes labelled SPIN1 in the cell lysates, probe **2** demonstrated a more robust labelling of SPIN1 with much higher ‘signal-to-noise’ (*i.e.*, specific-to-nonspecific) ratio when compared with probe **1** (Fig. 6a), revealing a better photo-cross-linking efficiency and specificity of the diazirine-based probe.

We then compared the abilities of probe **1** and probe **2** in the application of capturing endogenous H3K4Me_3_ readers from whole-cell lysates. To this end, we treated HeLa S3 cell lysates with probes **1**, **2** and **C1**, respectively. After UV irradiation, the cross-linked proteins were conjugated with biotin azide (biotin-N_3_) *via* click chemistry, followed by streptavidin enrichment. The enriched proteins were then resolved by SDS-PAGE and subjected to a western blot analysis using an antibody against endogenous ING2. As shown in Fig. 6b, while both probe **1** and probe **2** can capture endogenous ING2, the final ‘pull-down’ yield of probe **2** (∼8%) is four times higher than that of probe **1** (∼2%). This result further demonstrates the superiority of diazirine-based photoaffinity probes for identifying histone PTM readers in complex proteomes.

### Development of diazirine-based probes to identify ‘erasers’ for histone lysine acylations

**Fig. 7:**
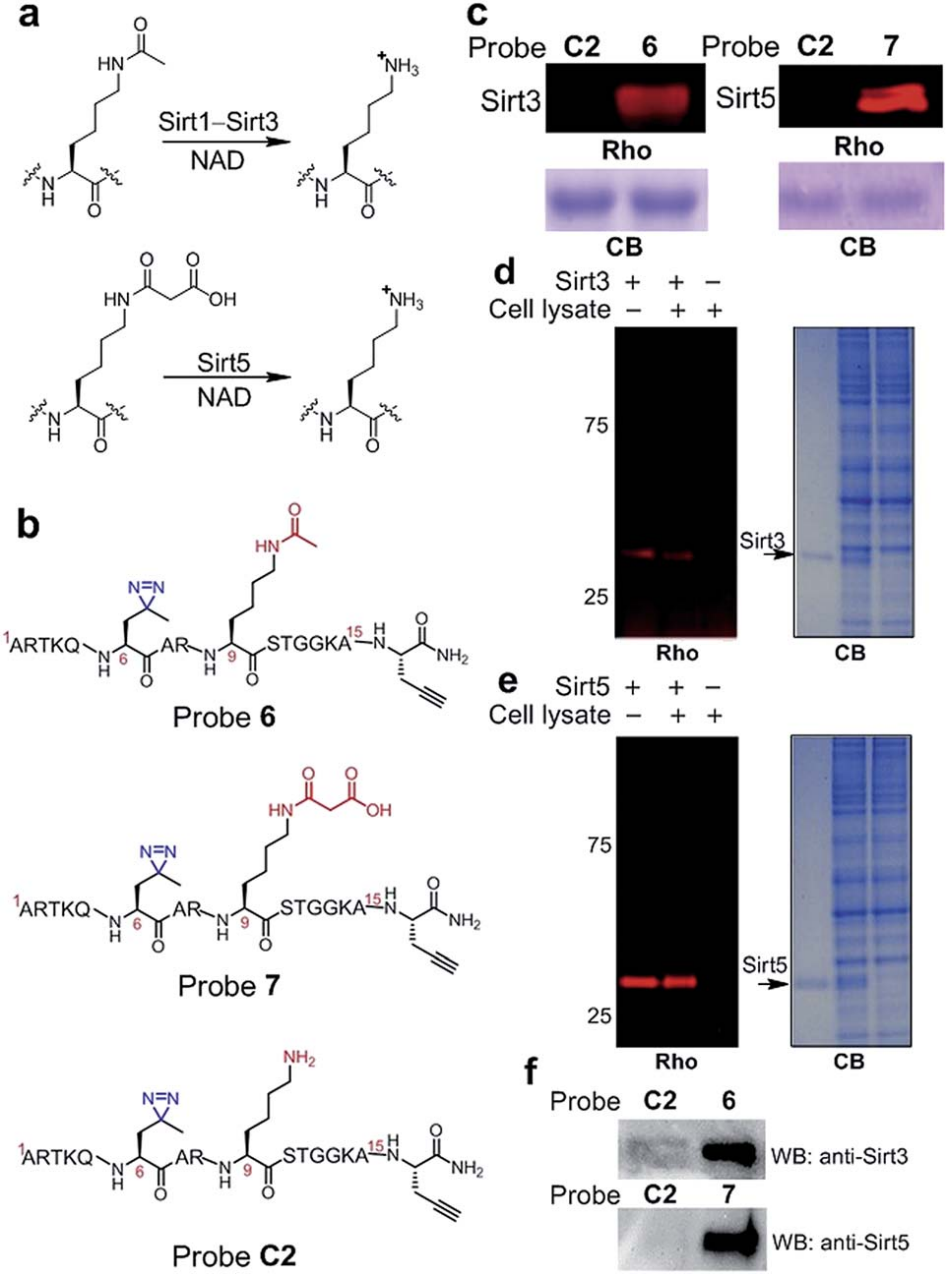
(a) Lysine deacetylation and demalonylation catalysed by Sirt1–Sirt3 and Sirt5, respectively. (b) Chemical structures of probes **6**, **7** and **C2**. (c) The labelling of Sirt3 (20 ng μL^–1^) and Sirt5 (20 ng μL^–1^) by probes **6** (2 μM) and **7** (2 μM), respectively. The specific labelling of recombinant (d) Sirt3 (20 ng μL^–1^) and (e) Sirt5 (20 ng μL^–1^) in HeLa S3 cell lysates (1.5 mg mL^–1^) by using probes **6** and **7** (2 μM), respectively. (f) Western blotting analysis shows the enrichment of endogenous Sirt3 and Sirt5 from the whole cell lysate by probes **6** and **7**, respectively. HeLa S3 whole cell lysate (3.0 mg mL^–1^) was incubated with the probes (2 μM) for 15 min. After UV irradiation (365 nm, 10 min), the labelled proteins were conjugated to biotin through click chemistry. Following enrichment by streptavidin beads, the enriched proteins were resolved by SDS-PAGE and analyzed by western blotting. Rho, rhodamine fluorescence; CB, Coomassie blue; WB: western blot.

Inspired by the high efficiency of the diazirine-based probes in the labelling of H3K4Me_3_ ‘readers’, we sought to test whether this photoreactive group could be used to develop chemical probes for the identification of more dynamic or transient interactors, such as enzymes that remove histone PTMs (*i.e.*, ‘erasers’). To develop this method, we focused on sirtuins, a family of enzymes known as nicotinamide adenine dinucleotide (NAD)-dependent protein deacetylases.^25^ There are seven human sirtuins called Sirt1–Sirt7. While Sirt1–Sirt3 demonstrate robust deacetylase activities, Sirt4–Sirt7 have very weak or undetectable deacetylase activities. However, recent studies have revealed that these sirtuins with weak deacetylase activities can function as deacylases for the removal of different acyl groups from protein lysine residues.^26^ For example, it was recently demonstrated that Sirt5 preferentially hydrolyzes malonyl and succinyl lysine (Fig. 7a).^27–29^ Based on this information, we designed probes **6** and **7** to examine their potential use for the identification of ‘erasers’ of histone lysine acetylation (*e.g.*, Sirt1–Sirt3) and malonylation (*e.g.*, Sirt5). These two probes are based on a histone H3 peptide (residues 1–15), where residue Lys 9 was acetylated and malonylated in probes **6** and **7**, respectively. In addition, photo-Leu was incorporated at the Thr 6 position as the photoreactive moiety, and propargyl glycine was attached at the C-termini for bioorthogonal conjugations in these probes (Fig. 7b).

To examine whether probes **6** and **7** can capture their corresponding ‘erasers’, we performed photo-cross-linking experiments using probes **6** and **7** to react with recombinant human Sirt3 and Sirt5, respectively. As shown in Fig. 7c, probes **6** and **7** successfully labelled Sirt3 and Sirt5, respectively, whereas an unmodified control probe **C2** failed to label either of these two enzymes. To analyse the labelling specificity, Sirt1, Sirt3 and Sirt5 were photo-cross-linked with probes **6** and **7**. Among the three sirtuins, only Sirt1 and Sirt3 were labelled by probe **6**, while probe **7** specifically labelled Sirt5 (Fig. S4†). This result agrees well with the fact that Sirt1 and Sirt3 are deacetylases while Sirt5 preferentially catalyses the hydrolysis of malonyl lysine. In addition, the specific labelling of recombinant Sirt3 and Sirt5 in complex proteomes was also achieved using **6** and **7**, respectively, in total cell lysates of human HeLa S3 cells (Fig. 7d and e).

Finally, we examined whether probes **6** and **7** could also be used to label endogenous Sirt3 and Sirt5, respectively, from whole cell lysates. Probes **6**, **7** and **C2** were each added to HeLa S3 lysates, followed by UV-radiation, click chemistry using biotin-N_3_, SDS-PAGE and western blot analysis using antibodies against Sirt3 and Sirt5. As shown in Fig. 7f, endogenous Sirt3 and Sirt5 were robustly enriched by probes **6** and **7**, respectively, whereas probe **C2** failed to capture these enzymes. Taken together, these data suggest that the photoaffinity probes can be used to ‘trap’ ‘erasers’ of histone lysine acylations from complex proteomes.

## Conclusions

We have developed diazirine-based photoaffinity probes and examined their ability to capture the ‘readers’ of histone lysine methylation as well as the ‘erasers’ of histone lysine acetylation and malonylation. When compared with previously reported benzophenone-based photoaffinity probes, these new probes demonstrated higher photo-cross-linking efficiencies and specificities in the tested systems. A key issue in the design of photoaffinity probes is to determine where a photoreactive group should be placed. Our study showed that to ensure robust labelling of histone PTM ‘readers’, diazirine, due to its small size, has more flexibility with respect to incorporation sites, while bulky benzophenone is likely to ‘bump’ against ‘readers’ if it is placed at a ‘wrong’ position. In addition, the small size of diazirine also enables it to be incorporated closer to PTM sites, which is expected to facilitate more efficient and specific labelling of proteins that recognize the PTMs. These advantages therefore make diazirine a better choice for the development of photoaffinity probes to identify histone PTM ‘readers’, particularly when structural information to guide the design of probes is lacking. Furthermore, we demonstrated that the diazirine-based probes can also be used to capture lysine deacetylases and demalonylase. This study has therefore broadened the scope of our photo-cross-linking strategy from finding histone PTM ‘readers’ to identifying dynamic and transient interactions between PTMs and their ‘erasers’. We anticipate to use this strategy in conjunction with SILAC technology to comprehensively profile ‘erasers’ of newly identified histone PTMs such as lysine crotonylation. These studies will be reported in due course.
