# Supplementary material for: Developing diazirine-based chemical probes to identify histone modification ‘readers’ and ‘erasers’
Source: Chem Sci. 2014 Nov 12;6(2):1011–7. doi: 10.1039/c4sc02328e (PMC5811097; doi:10.1039/c4sc02328e)
Supplement: Supplementary file 1 [file SC-006-C4SC02328E-s001.pdf]

Electronic Supplementary Material (ESI) for Chemical Science.
This journal is © The Royal Society of Chemistry 2014

Supporting Information

Developing diazirine-based chemical probes to identify histone modification 'readers' and 'erasers'

Tangpo Yang, Zheng Liu, Xiang David Li

Department of Chemistry,The University of Hong Kong, Pokfulam Road, Hong Kong, China.

Email: xiangli@hku.hk

List of Contents

Fig. S1. Dose-dependent labeling of SPIN1, ING2and MORC3 by probes **1**, **2** and **3**

Fig. S2. Quantification of labeling yields of SPIN1 by probes **1**, **2** and **3**

Fig. S3. The labeling of MORC3 by probes **4** and **5**

Fig. S4. The selective labelling of Sirt1 and Sirt3 by probe **6**, and Sirt5 by probe **7**

Fig. S5. LC-MS analysis of probe **1**

Fig. S6. LC-MS analysis of probe **2**

Fig. S7. LC-MS analysis of probe **3**

Fig. S8. LC-MS analysis of probe **4**

Fig. S9. LC-MS analysis of probe **5**

Fig. S10. LC-MS analysis of probe **6**

Fig. S11. LC-MS analysis of probe **7**

Fig. S12. LC-MS analysis of probe **C1**

Fig. S13. LC-MS analysis of probe **C2**

Experimental Methods

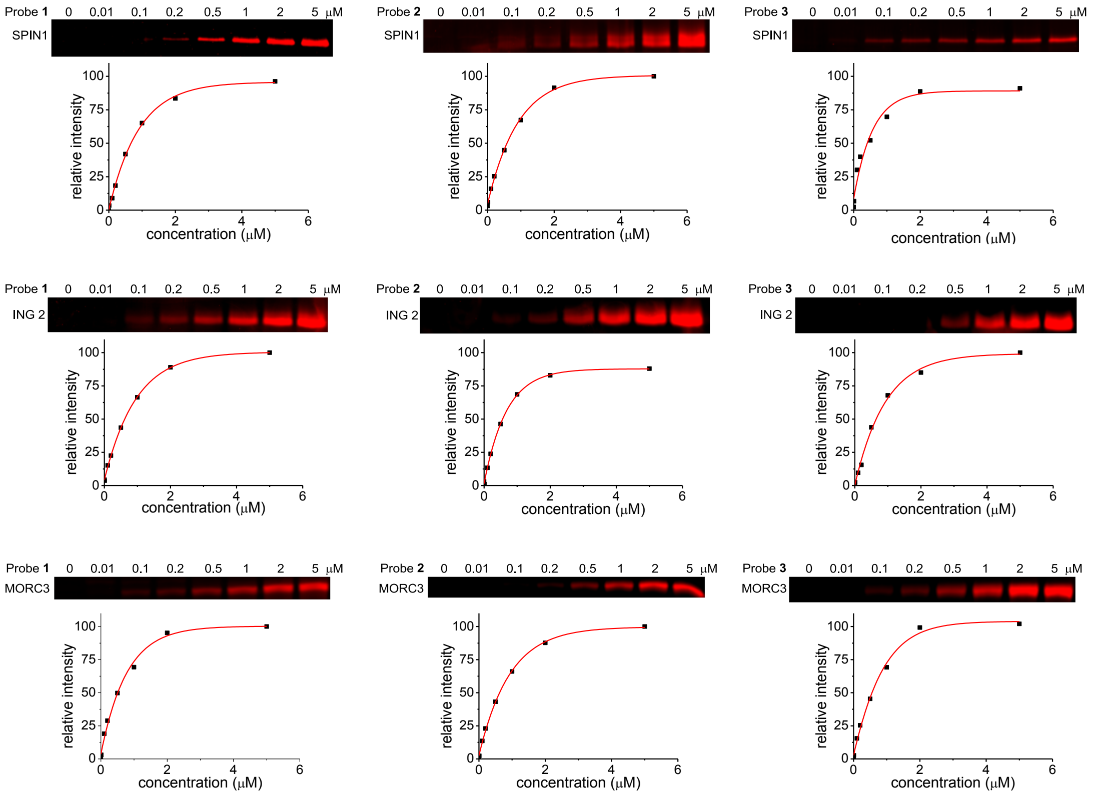

Fig. S1. The dose-dependent labeling of SPIN1, ING2 and MORC3by probes**1**, **2** and**3**.The recombinant proteins (20 ng/μL) was incubated with the indicated probes (2 μM), respectively. After UV irradiation (365 nm) for 1 hour, the labeled proteins were conjugated to rhodamine-azide, resolved by SDS-PAGE, and detected by in-gel fluorescence scanning.

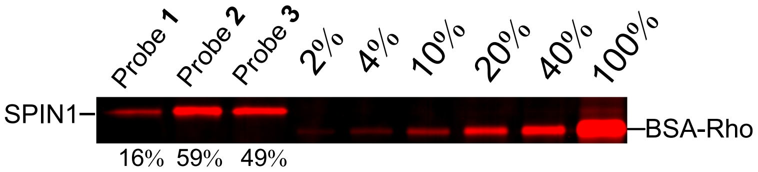

Fig. S2. The quantification of labeling yields of SPIN1 by probes**1**,**2** and **3**.Recombinant SPIN1(20 ng/μL) was incubated with probes (2 μM) **1**, **2**and **3**. After UV irradiation (365 nm) for 1 hour, the labeled proteins were conjugated to rhodamine-azide, resolved by SDS-PAGE, and detected by in-gel fluorescence scanning. A series amount of rhodamine-conjugated BAS (Rho-BSA) was loaded on the same gel for the quantification of the labeling yields of SPIN1 by the different probes.

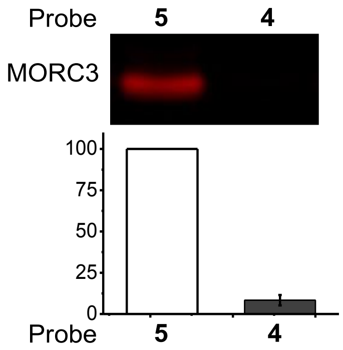

Fig. S3. The labeling of MORC3(20 ng/μL) by probes **4** and **5** (2 μM). The protein was photo-labeled (365 nm) with probes **4** for 1 h, and with probe **5** for 20 min, respectively. The labeled proteins were conjugated to rhodamine-azide and detected by in-gel fluorescence scanning. The column chart shows the relative fluorescence intensity of the labeled proteins bands. The fluorescence intensities of probe**5** labeled proteins was set to 100%. Data are averages ± s.e. (n= 2).

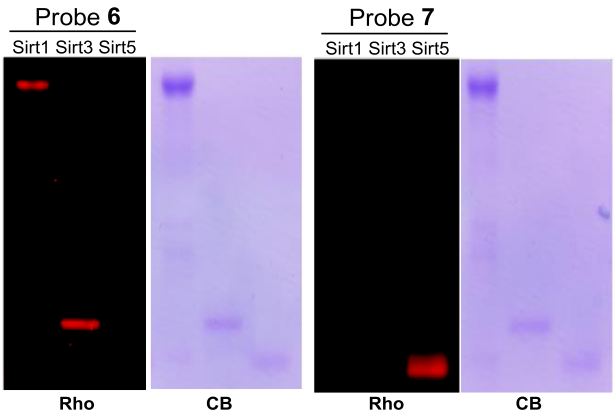

Fig. S4. The selective labelling of (left) Sirt1 and Sirt3 by probe **6** (2 μM), and (right) Sirt5 by probe **7** (2 μM), in a mixture of Sirt1 (60 ng/μL), Sirt3 (20 ng/μL) and Sirt5 (20 ng/μL). Rho, Rhodamine fluorescence;CB, Coomassie blue.

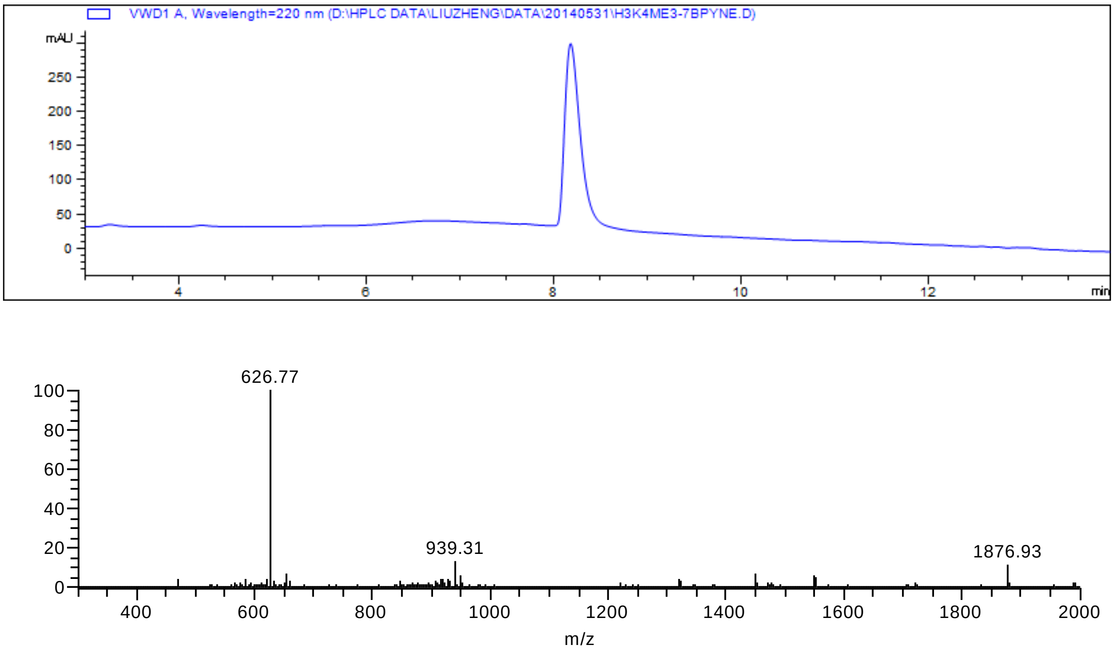

Fig. S5. LC–MS analysis of probe **1**. *m/z*1876.93 (M +H$^+$), calculated *m/z* 1877.05 (M +H$^+$); *m/z* 939.31 (M + 2H$^+$), calculated *m/z* 939.02 (M + 2H$^+$); *m/z* 626.77 (M + 3H$^+$), calculated *m/z* 626.35 (M + 3H$^+$);

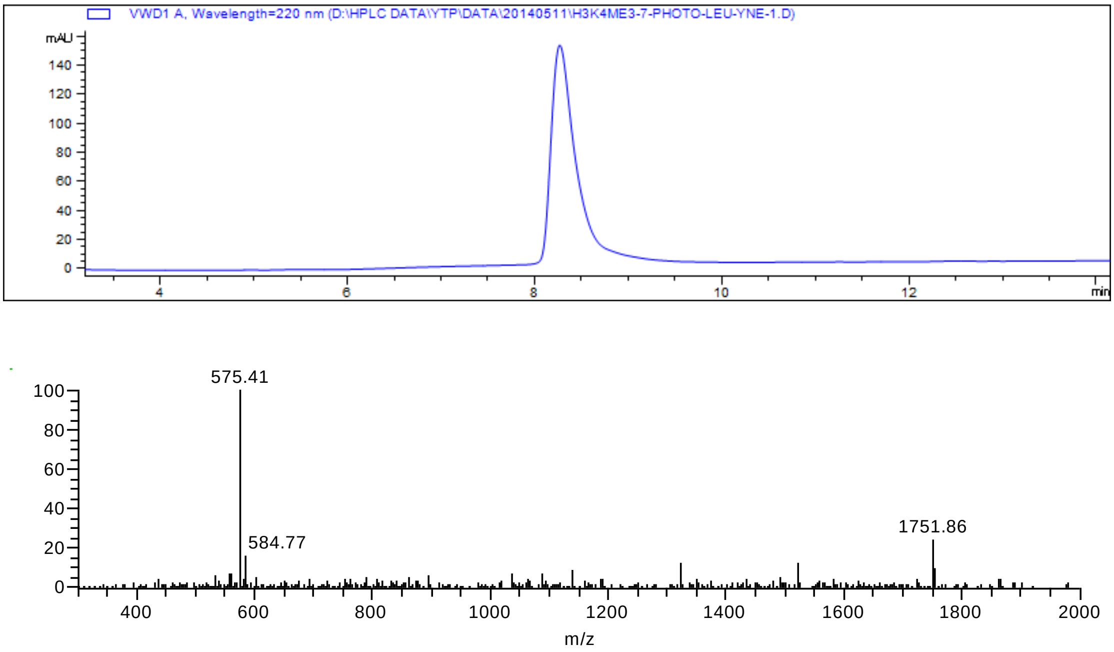

Fig. S6. LC–MS analysis of probe **2**. *m/z*1751.86 (M + 1H$^+$), calculated *m/z* 1751.0147 (M + 1H$^+$); *m/z* 584.77 (M + 3H$^+$), calculated *m/z* 584.34 (M + 3H$^+$); *m/z* 575.41 (M– N_2+ 3H$^+$), calculated *m/z* 575.00 (M– N_2+ 3H$^+$)

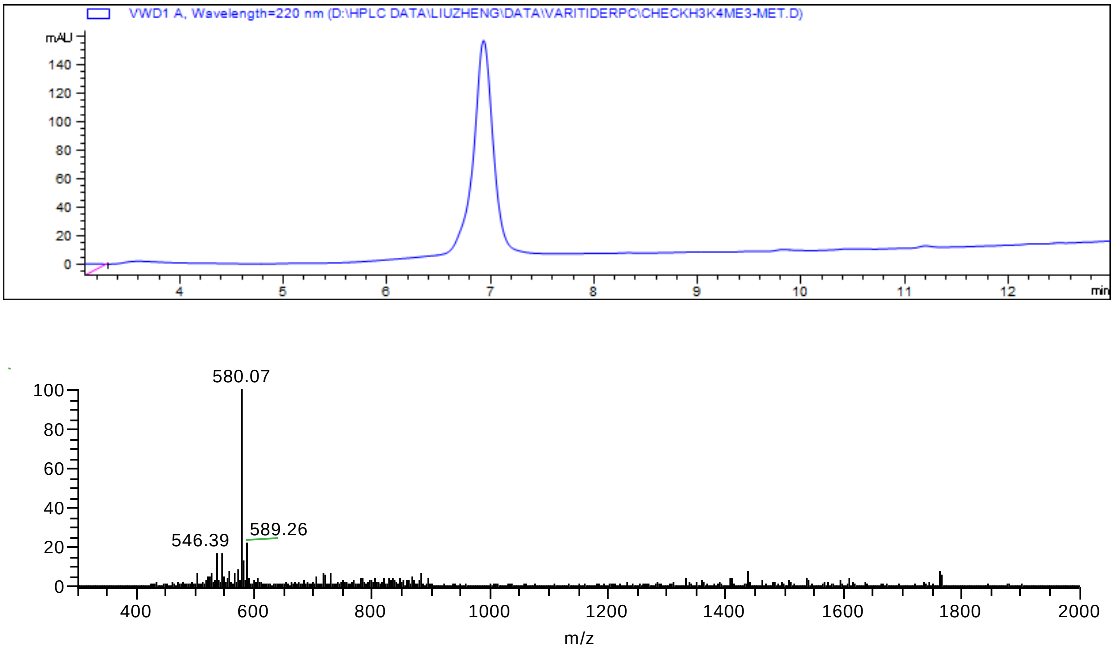

Fig. S7. LC–MS analysis of probe **3**. *m/z*589.26 (M + 3H$^+$), calculated *m/z* 589.01 (M + 3H$^+$); *m/z* 580.07 (M– N_2+ 3H$^+$), calculated *m/z* 580.01 (M– N_2+ 3H$^+$);

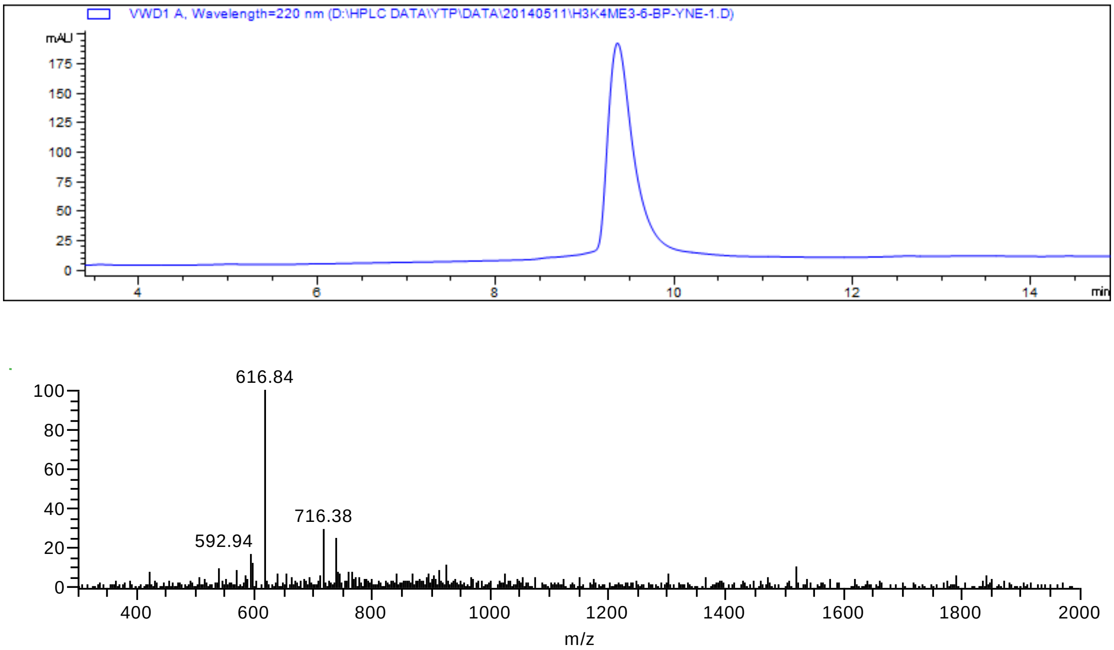

Fig. S8. LC–MS analysis of probe **4**. *m/z*616.84 (M + 3H$^+$), calculated *m/z* 616.34 (M + 3H$^+$)

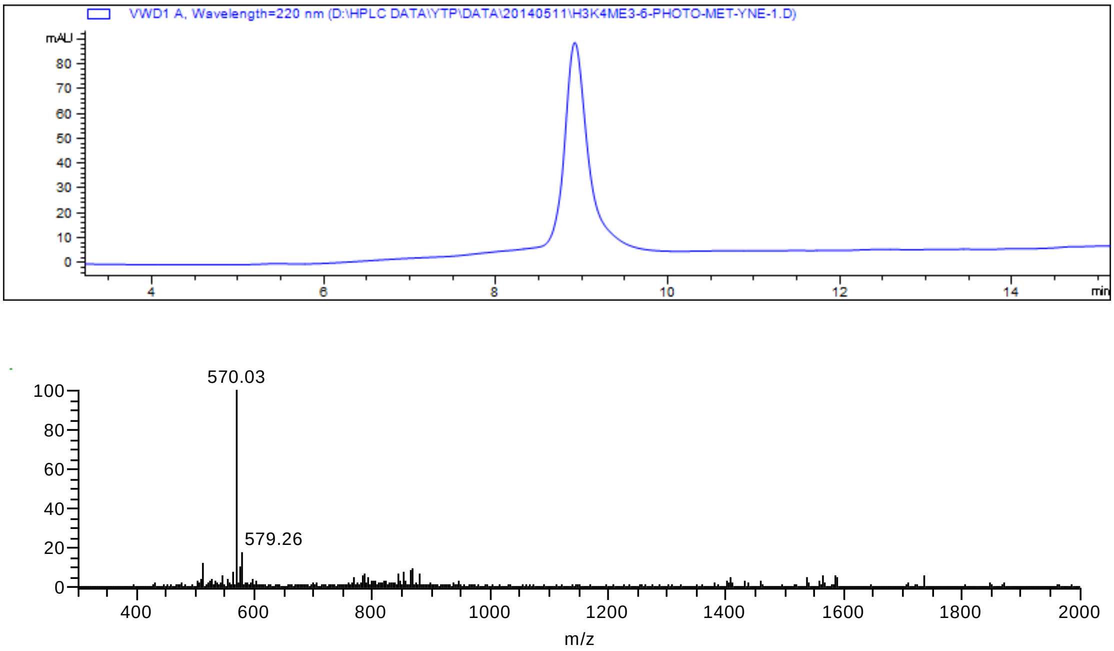

Fig. S9. LC–MS analysis of probe **5**. *m/z*579.26 (M + $3H^+$), calculated *m/z* 579.01 (M + $3H^+$); *m/z* 570.03 (M–N_2 + $3H^+$), calculated *m/z* 569.67 (M–N_2 + $3H^+$);

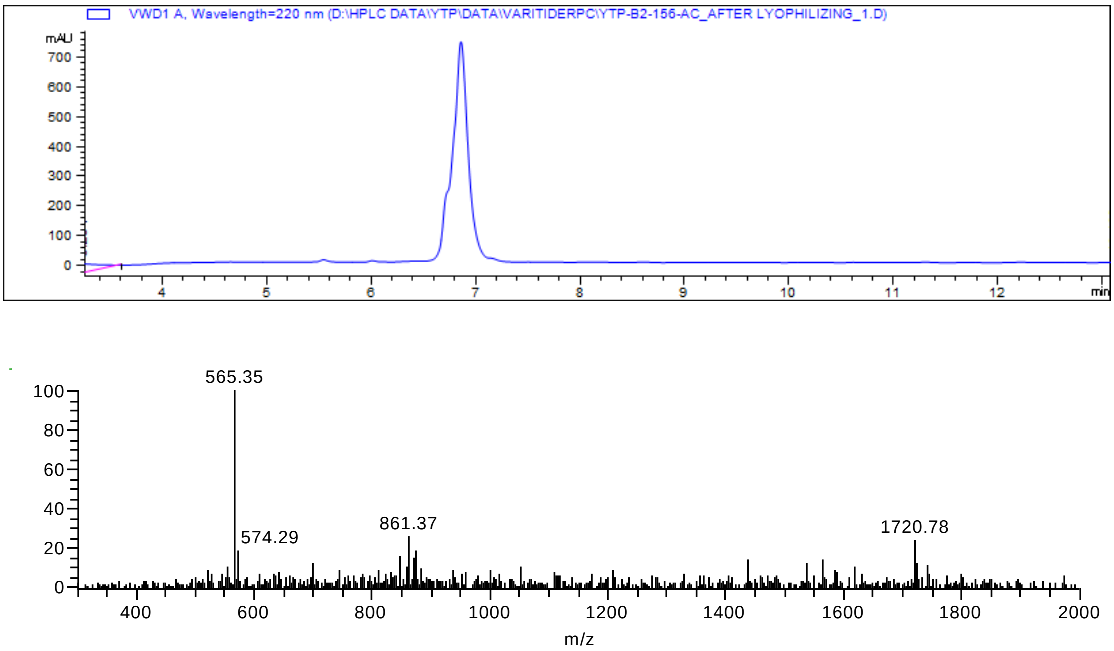

Fig. S10. LC–MS analysis of probe **6**. *m/z* 1720.78 (M + H^+), calculated *m/z* 1720.96 (M + H^+); *m/z* 861.37 (M+$2H^+$), calculated *m/z* 860.98 (M+$2H^+$); *m/z* 574.29 (M +$3H^+$), calculated *m/z* 574.32 (M +$3H^+$); *m/z* 565.35 (M–N_2 +$3H^+$), calculated *m/z* 564.99 (M–N_2+$3H^+$);

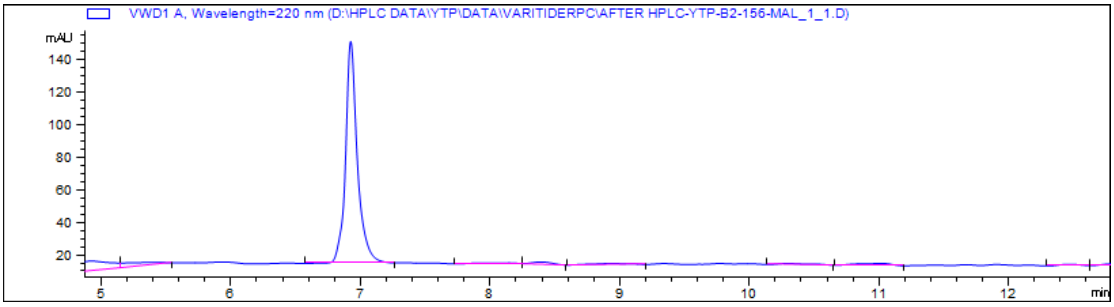

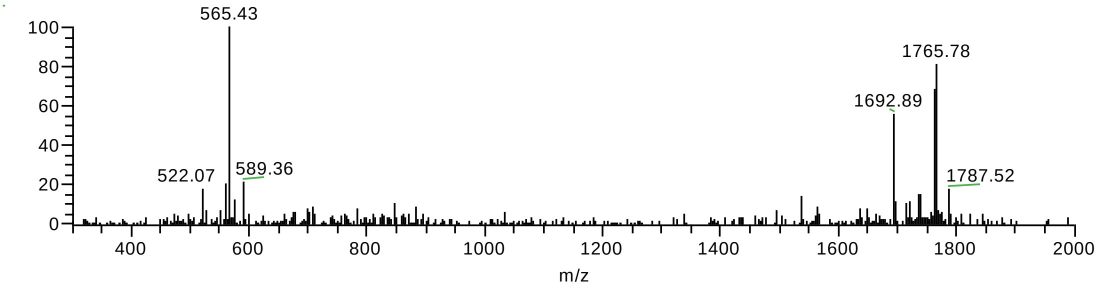

Fig. S11. LC–MS analysis of probe **7**.*m/z* 1765.78 (M + H$^+$), calculated *m/z* 1764.95 (M + H$^+$); *m/z* 1692.89 (M–CO_2–N_2+ H$^+$), calculated *m/z* 1692.95 (M–CO_2–N_2+ H$^+$); *m/z* 589.36 (M +3H$^+$), calculated *m/z* 588.98 (M +3H$^+$); *m/z* 565.43 (M–CO_2–N_2+3H$^+$), calculated *m/z* 564.98 (M–CO_2–N_2+3H$^+$);

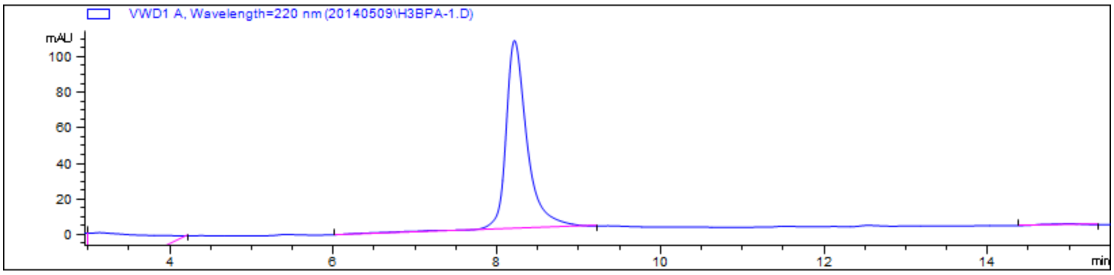

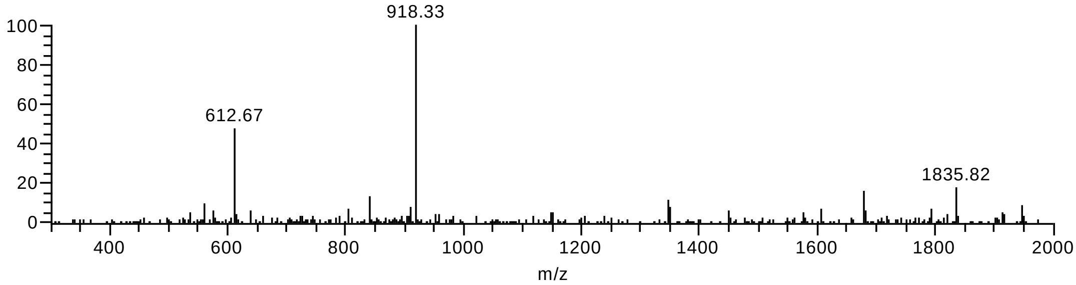

Fig. S12. LC–MS analysis of probe **C1**. *m/z*1835.82 (M + H$^+$), calculated *m/z* 1834.99 (M + H$^+$); *m/z* 918.33 (M+2H$^+$), calculated *m/z* 917.99 (M + 2H$^+$); *m/z* 612.67 (M +3H$^+$), calculated *m/z* 612.33 (M +3H$^+$)

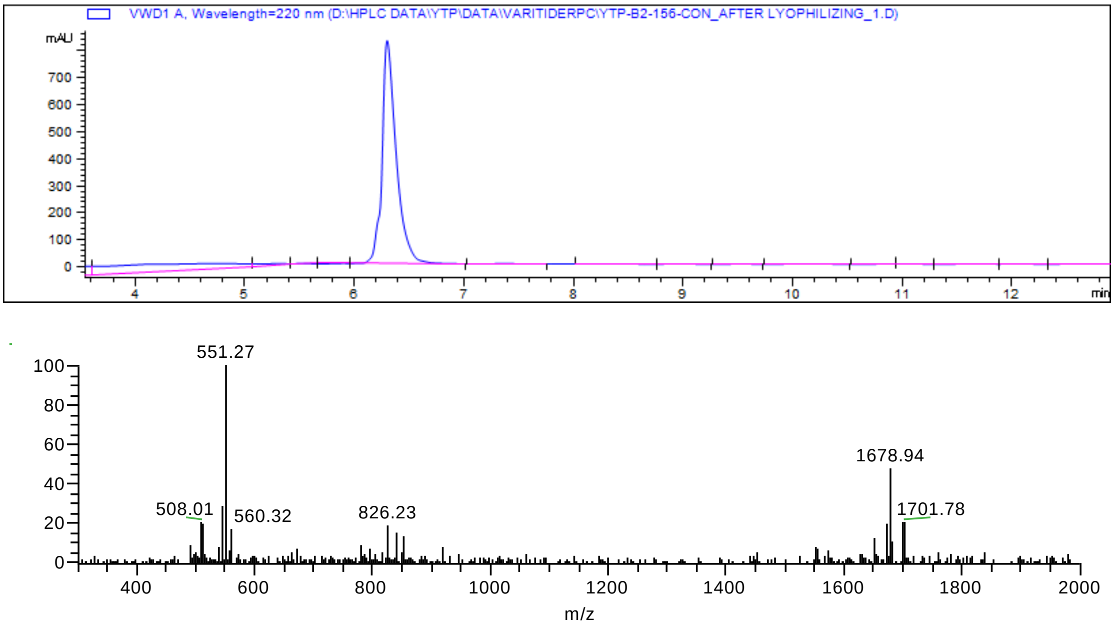

Fig. S13. LC–MS analysis of probe **C2**. *m/z* 1678.94 ($M + H^+$), calculated *m/z* 1678.95 ($M + H^+$); *m/z*826.23 ($M– N_2$+$2H^+$), calculated *m/z* 825.97 ($M– N_2$+$2H^+$); *m/z* 560.32 (M+$3H^+$), calculated *m/z* 560.32(M+$3H^+$);*m/z* 551.27 ($M– N_2$+$3H^+$), calculated *m/z* 550.98($M– N_2$+$3H^+$);

Experimental Methods

General Methods and Materials

Unless otherwise noted, all the chemicals reagents were purchased from Sigma–Aldrich. NMR spectra were recorded on a Bruke UltraShield 400 MHz spectrometer and were calibrated using residual undeuterated solvent as an internal reference. Chemicals shifts were reported in values (ppm). Peptides were purified on a preparative high performance liquid chromatography (HPLC) system with Waters 2535 Quaternary Gradient Module, Waters 515 HPLC pump, Waters SFO system Fluidics Organizer and Waters 2767 Sample Manager.

Photo-cross-linking experiments

The benzophenone-containing probes or diazirine-containing probes were incubated with protein (20 μg/mL) or whole cell proteome samples (1.5mg/mL or 3 mg/mL) in the buffer(50mM4-(2-hydroxyethyl)-1-piperazineethanesulfonic acid (HEPES), 150 mM NaCl, 2 mM $MgCl_2$, 0.1% tween-20, 20% glycerol, pH 7.5) for 10min at 4°C. Then, the samples were irradiated at 365 nm using UV lamp for 20 or 60 min.

Cu(I)-Catalyzed Cycloaddition/Click Chemistry

The procedure was modified from Yang, Y. *et al. Chem. Biol.* 2010, 17, 1212. Briefly, after UV cross-linking, rhodamine-azide for in-gel fluorescence scanning or cleavable biotin azide for streptavidin enrichment was addedat final concentration of 100 μM, followed by tris(2-carboxyethyl)phosphine (TCEP)(1 mM), tris[(1-benzyl-1H-1,2,3-triazol-4-yl)methyl]amine (TBTA) (100 μM)and finally the reactions were initiated by the addition of $CuSO_4$ (1 mM). The reactions were incubated for 1 h at room temperature.

In-gel Fluorescence Visualization

The click chemistry reactions were quenched by adding 1 volume of 2X loading buffer. The proteins were heated at 85 ^{o}C for 8 min, and resolved by sodium dodecyl sulfate-polyacrylamide gel electrophoresis (SDS-PAGE). The labeled proteins were visualized by scanning the gel on a Typhoon 9410 variable mode imager (excitation 532 nm, emission filter 580 nm).

Streptavidin affinity enrichment of biotinylated proteins

After the click chemistry with cleavable biotin azide, the reaction was quenched by adding 4 volumes of ice-cold acetone to precipitate proteins. After washing with ice-cold methanol twice, the air-dried protein pellet was dissolved in PBS with 4% SDS, 20 mM EDTA and 10% glycerol by vortexting and heating. The solution was then diluted with PBS to give a final concentration of SDS of 0.5%. High-capacity streptavidin agarose beads (Thermo Pierce) were added to bind the biotinylated proteins with rotating for 1.5 hrs at room temperature. To remove nonspecific binding, the beads were washed with PBS with 0.2% SDS, 6 M urea in PBS with 0.1% SDS and 250 mM NH_4HCO_3 with 0.05% SDS. The enriched proteins were then eluted by incubating with 25 mM Na_2S_2O4, 250 mMNH_4HCO_3 and 0.05% SDS for 1 hr. The eluted proteins were dried down with SpeedVac.

Western blot.

Proteins separated by SDS-PAGE were transferred onto a PVDF membrane which was then

blocked (5% nonfat dried milk and 0.1% tween-20 in PBS) for 1 hour at room temperature. The membrane was incubated with primary antibody diluted in PBST (0.1% Tween-20 in PBS) with 2% BSA, followed by washing with PBST for 5min trice, incubated with goat anti-rabbit-HRP conjugated secondary antibody (1:10000, Santa Cruz), or rabbit anti-mouse-HRP conjugated secondary antibody (1:5000, Santa Cruz) diluted in PBST for 1hr at room temperature, and then visualized with western blotting detection reagents (Thermo).

Protein expression and purification

Plasmids of Sirt1 (193-747) and Sirt5 (34-302) for*E. coli* expression were generated as previously described.[1,2] Plasmids of Sirt3 (102-399) cloned in pTrcHis 2C vector for *E.coli* expression was generous gift from Eric Verdin (UCSF). All the proteins were expressed in *E.coli* Rosetta cells.

<u>Sirt1 expression and purification</u>

The protein was expressed in *E.coli* Rosetta cells. To induce the expression of Sirt3, isopropyl-d-1 thiogalactopyranoside (IPTG) was added to a final concentration of 0.1 mM when OD600 was 0.6, and the culture was grown at 15 ^{o}C for another 16 h. Cells were harvested and then resuspended in lysis buffer (50 mM Tris-HCl, pH 7.5, 500 mMNaCl, 1 mM TCEP). Following sonication and centrifugation, the supernatant was loaded onto a nickel column pre-equilibrated with lysis buffer. The column was washed with 5 column volumes of wash buffer (50 mMTris-HCl, pH 7.5, 500 mM NaCl, 30 mM Imidazole) and then target protein was eluted with elution buffer (50 mMTris-HCl, pH 7.5, 500 mMNaCl, 250 mM Imidazole). The protein was further loaded onto a Highload 26/60 Superdex200 gel-filtration column (GE Healthcare Life Sciences) and was eluted with 50 mM Tris-HCl, pH 7.5, 500 mMNaCl and 2 mM DTT. After concentration, the target protein was frozen and then stored at –80 ^{o}C for later use.

<u>Sirt3 expression and purification</u>

Sirt3 was expressed in *E.coli* Rosetta cells. To induce the expression of Sirt3, IPTG was added to a final concentration of 0.2 mM when OD600 was 0.8, and the culture was grown at 18 ^{o}C overnight. Cells were harvested and then resuspended in lysis buffer (50 mM HEPES, pH 7.5, 150 mMNaCl, 1 mM TECP). Following sonication and centrifugation, the supernatant was loaded onto a nickel column pre-equilibrated with lysis buffer. The column was washed with 5 column volumes of wash buffer (50 mM HEPES, pH 7.5, 150 mMNaCl, 1 mM TCEP, 30 mM Imidazole) and then target protein was eluted with elution buffer (50 mM HEPES, pH 7.5, 150 mM NaCl, 1 mM TCEP, 250 mM Imidazole). The protein was further loaded onto a Highload 26/60 Superdex75 gel-filtration column (GE Healthcare Life Sciences) and eluted with 20 mM Tris-HCl, pH 7.5, 150 mMNaCl, 2 mM DTT. After concentration, the target protein was frozen and then stored at –80 ^{o}C for later use.

<u>Sirt5 expression and purification</u>

Sirt5 was expressed in *E.coli* Rosetta cells. To induce the expression of Sirt5, IPTG was added to a final concentration of 0.2 mM when OD600 was 0.8, and the culture was grown at 18 ^{o}C overnight. Cells were harvested and then resuspended in lysis buffer (50 mM HEPES, pH 7.5, 500 mM NaCl, 1 mM TECP). Following sonication and centrifugation, the supernatant was loaded onto a nickel column pre-equilibrated with lysis buffer. The column was washed with 5 column volumes of wash buffer (50 mM HEPES, pH 7.5, 500 mM NaCl, 1 mM TCEP, 30 mM Imidazole) and then

target protein was eluted with elution buffer (50 mM HEPES, pH 7.5, 500 mM NaCl, 1 mM TCEP, 250 mM Imidazole). The protein was further loaded onto a Highload 26/60 Superdex75 gel-filtration column (GE Healthcare Life Sciences) and was eluted with 20 mM Tris-HCl, pH 7.5, 150 mM NaCl, 2 mM DTT. After concentration, the target protein was frozen and then stored at −80 °C for later use.

The GST-fusion protein expression and purification.

The PHD finger of ING2 (residues 208-270), full length mouse SPIN1 were cloned into pGex-6p-1 vector with an N-terminal glutathione S-transferase (GST) tag.Gateway cloning technology (Invitrogen) was used to clone the ZW finger of MORC3 (residues 400−461) into pDONR201 and pDEST15, with a prescission protease site added to the N-terminus of the open reading frame.

Recombinant GST-tagged mouse SPIN1, human ING2 PHD finger and human MORC3 ZW finger were expressed in *E.coli* Rosetta cells. The expression of GST-tagged proteins were induced by adding 0.5 mM IPTG when OD600 reached to 0.8, and the culture was grown at 18 °C overnight. Cells were harvested and then resuspended in lysis buffer (50 mM HEPES, pH 7.5, 150 mMNaCl, 2 mM DTT). Following sonication and centrifugation, the supernatant was incubated with Glutathione Sepharose 4B beads pre-equilibrated with resuspension buffer for 90 min. Then, wash the beads with lysis buffer for 3 times. The target protein can be eluted with elution buffer (50 mM HEPES, pH 7.5, 150 mM NaCl, 2 mM DTT, 25 mM Glutathione). After dialysis to lysis buffer and concentration, the GST-tagged proteins were frozen and stored at −80 °C for later use.

Peptide synthesis.

Fmoc-L-propargylglycine (Fmoc-Pra-OH), Fmoc-4-benzoyl-L-phenylalanine (Fmoc-Bpa-OH) and Fmoc-N_ε-(trimethyl)-L-lysine chloride (Fmoc-Lys(Me_3)-OH) were purchased from Chem-Impex International (Wood Dale, IL). All peptides used in this research were synthesized on Rink-Amide MBHA resin followed standard Fmoc-based solid-phase peptide synthesis protocol.

After the coupling of all amino acids, the removal of protecting groups and cleavage of peptides from the resin were done by incubating the resin with cleavage cocktail containing 95% trifluoroacetic acid (TFA), 2.5% triisopropylsilane, 1.5% water and 1% thioanisole for 2 h. Peptides were purified by preparative HPLC with an XBridge Prep OBDTM C18 column (30 mm 250 mm, 10 μm, Waters). Mobile phase used were water with 0.1% TFA (buffer A) and 90% acetonitrile (ACN) in water with 0.1% TFA (buffer B). The purity (> 95%) and identity of peptides were confirmed by LC-MS (Figure S3-S11). HPLC runs used linear gradients of 0.05% TFA in water (solvent A) and 0.05% TFA 90% acetonitrile in water (solvent B).

Synthesis of Fmoc-photo-Met and Fmoc-photo-Leu.

Scheme 1. Synthesis of Fmoc-photo-Met

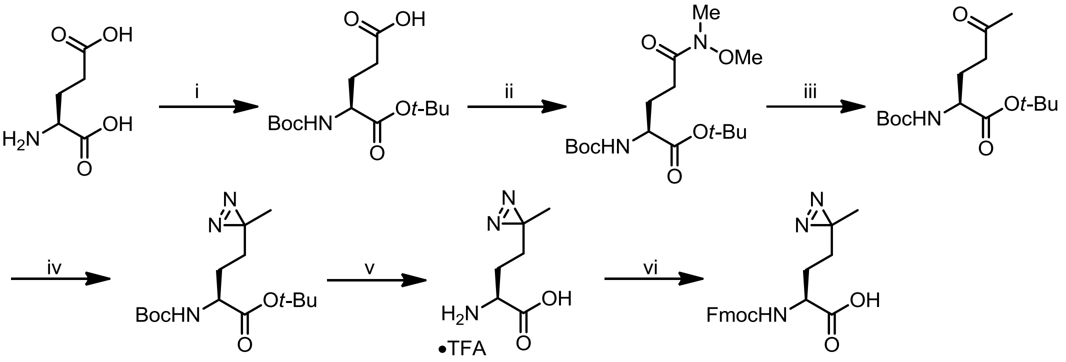

Conditions: (i) (a) TMSCl, MeOH, room temperature, quantitative yield; (b)Boc_2O, $NaHCO_3$, 1,4-dioxane/H_2O (2:1), 0 °C to room temperature, 99% yield; (c) *t*-BuOH, DCC, DMAP, 0°C to room temperature, 64% yield; (d)LiOH, THF/H_2O (2:1), room temperature 82% yield; (ii) *N*-methylmorpholine, isobutyl chloroformate, *N,O*-dimethylhydroxylamine, DCM, 99% yield; (iii)MeMgBr, toluene, 61% yield; (iv) (a) NH_3, hydroxylamine-*O*-sulfonic acid; (b) TEA, I_2, MeOH, 54% yield; (v) 4M HCl, THF,quantitative yield; (vi) Fmoc-OSu, $NaHCO_3$, 1,4-dioxane/H_2O, 99% yield.

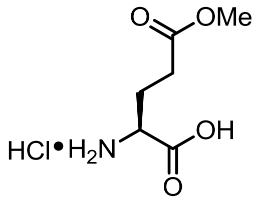

(S)-2-amino-5-methoxy-5-oxopentanoic acid hydrochloride (H-Glu(OMe)-OH)[3] Trimethylsilyl chloride (28 mL, 2.2 equiv) was dropped into the suspension of L-glutamic acid (14.7 g, 100 mmol) in methanol over 5 min under argon. After stirring at room temperature for 20 min, the solvent was removed by evaporation. A white solid (20.52 g, quantitative yield) was obtained and used for the next step without any purification.^{1}H NMR (400 MHz, MeOD): 4.06 (t, *J* = 6.60 Hz, 1H), 3.70 (s, 3H), 2.63-2.56 (m, 2H), 2.27-2.13 (m, 2H) ppm;

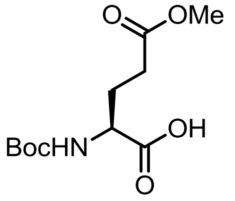

(S)-2-((*tert*-butoxycarbonyl)amino)-5-methoxy-5-oxopentanoic acid (Boc-Glu(OMe)-OH)[3]H-Glu(OMe)-OH (19.76 g, 100 mmol) was dissolved in water (100 mL) and dioxane (200 mL) at 0 °C. Then Boc_2O (26.19 g, 1.2 equiv) and $NaHCO_3$ (21.00 g, 2.5 equiv) were added to the solution and stirred at room temperature overnight. After removing the dioxane by rotavapor, the aqueous solution was washed by diethyl ether. Then 1M HCl was used to adjust the pH to be around 3 to 4. The suspension was extracted with ethyl acetate. The combined

organic solution was washed with water and brine, respectively; dried over Na_2SO_4. The Boc-Glu(OMe)-OH (liquid, 26.8 g, 99% yield) was obtained by evaporating all the solvent and used for the next step without further purification.^{1}H NMR (400 MHz, $CDCl_3$):5.21 (d, *J* = 6.98 Hz, 1H), 4.35 (d, *J* = 5.22 Hz, 1H), 3.69 (s, 3H), 2.53-2.44 (m, 2H), 2.27-2.22 (m, 1H), 2.03-1.99 (m, 1H), 1.45 (s, 9H) ppm;

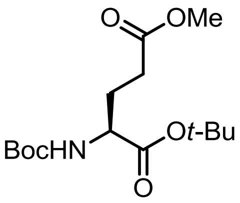

(S)-1-*tert*-butyl-5-methyl-2-((*tert*-butoxycarbonyl)amino)pentanedioate(Boc-Glu(OMe)-O*t*-Bu)[3]DCC (20.63 g, 1.2 equiv), DMAP (1.02 g, 10 mol%) and *t*-BuOH (79 mL, 10.0 equiv) were added into ice-cold DCM. Then a solution of Boc-Glu(OMe)-OH (21.77 g, 83.32 mmol) in DCM was dropped into the mixture over 30 min. The mixture was stirred at 0 °C for 1h and then at room temperature overnight. The suspension was filtered through Celite® to remove DCU. And the organic solution was washed with 0.1 M HCl solution, $NaHCO_3$ (sat.) and brine, respectively; dried over Na_2SO_4. The solution was concentrated and the product Boc-Glu(OMe)-Ot-Bu (17 g, 64% yield) was obtained by flash column chromatography as a white solid.^{1}H NMR (400 MHz, $CDCl_3$): 5.10 (d, *J* = 7.35 Hz, 1H), 4.20 (d, *J* = 5.08 Hz, 1H), 3.68 (s, 3H), 2.46-2.36 (m, 2H), 2.16-2.13 (m, 1H), 1.95-1.89 (m, 1H), 1.47 (s, 9H), 1.44 (s, 9H) ppm;

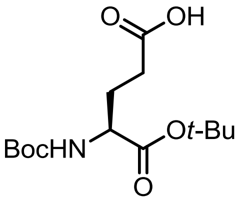

(S)-5-(*tert*-butoxy)-4-((*tert*-butoxycarbonyl)amino)-5-oxopentanoic acid (Boc-Glu(OH)-O*t*-Bu)[3] Boc-Glu(OMe)-O*t*-Bu (14.0 g, 44.11 mmol) was dissolved in THF (200 mL), then 1 M aqueous solution of LiOH (2.11 g, 2.0 equiv) was dropped over 30 min. After stirring at room temperature for 1h, the solution was cooled to 0 °C and 0.1 M HCl was added to adjust pH to be 3 to 4. The suspension was extracted with ethyl acetate. The combined organic layer was washed with brine and dried over Na_2SO_4. The white solid (11.01 g, 82% yield) was obtained by evaporating all the solvent and used for next step without further purification.^{1}H NMR (400 MHz, $CDCl_3$):5.17 (d, *J* = 6.92 Hz, 1H), 4.23 (d, *J* = 4.64 Hz, 1H), 2.50-2.41 (m, 2H), 2.18-2.16 (m, 1H), 1.91 (m, 1H), 1.47 (s, 9H), 1.45 (s, 9H) ppm;

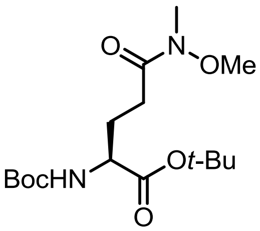

(S)-*tert*-butyl 2-((*tert*-butoxycarbonyl)amino)-5-(methoxy(methyl)amino)-5-oxopentanoate[4]. To a solution ofBoc-Glu(OH)-O*t*-Bu (4.6 g, 15.16 mmol) in anhydrous DCM was added N-methylmorpholine (4.17 mL, 2.5 equiv) followed by isobutyl chloroformate (2.56 mL, 1.3 equiv) at 0 °C. After stirring for 1h, the *N*,*O*-dimethylhydroxylamine hydrochloride (1.76 g, 1.2 equiv) was added portion wise. Then the mixture was stirred at room temperature overnight. The reaction was quenched by adding 0.1 M HCl. The mixture was extracted with DCM.Then the combined organic layer was washed with brine and dried over Na_2SO_4. The solution was concentrated and the white solid (5.25 g, 99% yield) was obtained by flash column chromatography.^{1}H NMR (400 MHz, $CDCl_3$):5.19 (d, J = 7.60 Hz, 1H), 4.19 (d, J = 4.70 Hz, 1H), 3.68 (s, 3H), 3.18 (s, 3H), 2.56-2.48 (m, 2H), 2.16-2.14 (m, 1H), 1.97-1.92 (m, 1H), 1.47 (s, 9H), 1.44 (s, 9H) ppm;

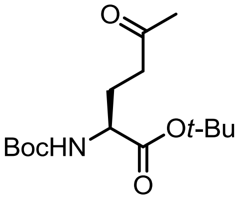

(S)-*tert*-butyl 2-((*tert*-butoxycarbonyl)amino)-5-oxohexanoate[4]To a solution of Weinreb amide (5.25 g, 15.18 mmol) in anhydrous toluene (50 mL) was dropped methylmagnesium bromide (3M in diethyl ether, 12 mL, 2.3 equiv) over 30 min at –78 °C. Then the reaction was warmed to –5 °C over 3 h and quenched with 0.1 M HCl. The mixture was extracted with ethyl acetate and dried over Na_2SO_4. The solution was concentrated and the white solid (2.8 g, 61% yield) as obtained by flash column chromatography.^{1}H NMR (400 MHz, $CDCl_3$): 5.07 (s, 1H), 4.14 (s, 1H), 2.60-2.46 (m, 2H), 2.15 (s, 3H), 2.10-2.09 (m, 1H), 1.86-1.81 (m, 1H), 1.47 (s, 9H), 1.44 (s, 9H) ppm;

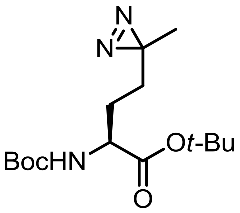

(S)-*tert*-butyl 2-((*tert*-butoxycarbonyl)amino)-4-(3-methyl-3H-diazirin-3-yl)butanoate[4]Ketone (2.15 g, 2.13 mmol) was dissolved in about 50 mL of liquid ammonia by ice-acetone condenser. The solution was stirred for 5 h at –30 °Cto–40 °C. Then the reaction flask was cooled to –50 °C and a solution of hydroxylamine-*O*-sulfonic acid (0.93 g, 1.2 equiv) in anhydrous methanol was dropped into the mixture over 30 min. The reaction was stirred at –30 °C to–40 °C for another 10 h

and then allowed to room temperature. Then ammonia was evaporated and the suspension was filtered through Celite® to remove the solid. The filter cake was washed with several portions of anhydrous methanol. The combined washings were concentrated until no smell of ammonia could be detected. The oil was re-dissolved in 10 mL of anhydrous methanol and cooled to 0 ^{o}C. TEA (2.0 equiv) was added followed by addition of iodine solution in MeOH until a brown color persisted. The reaction was stirred at room temperature for 1h, then concentrated and re-dissolved in ethyl acetate. The organic solution was washed by water and brine, respectively; dried over Na_2SO_4. The organic solution was concentrated and the white solid (1.2 g, 54% yield) was obtained by flash column chromatography.^{1}H NMR (400 MHz, $CDCl_3$): 5.00 (d, *J* = 6.90 Hz, 1H), 4.14 (s, 1H), 1.70-1.69 (m, 1H), 1.52-1.40 (m, 2H), 1.45 (s, 9H), 1.44 (s, 9H), 1.40-1.29 (m, 1H), 1.01 (s, 3H) ppm;

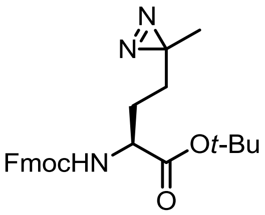

(S)-*tert*-butyl 2-((((9H-fluoren-9-yl)methoxy)carbonyl)amino)-4-(3-methyl-3H-diazirin-3-yl) butanoate Boc-protected diazirine (300 mg, 0.95 mmol) was dissolved in 30 mL of THF followed by adding 30 mL of 4M HCl. The mixture was stirred at room temperature until all the starting materials were consumed. Then all the solvents were removed and the white solid was obtained with quantitative yield. It was used for the next step without further purification. ^{1}H NMR (400 MHz, D_2O): δ = 4.02 (t, *J* = 6.40 Hz, 1H), 1.88-1.78 (m, 2H), 1.54-1.44 (m, 2H), 1.00 (s, 3H) ppm. The white solid was dissolved in water (5 mL) and dioxane (10 mL) followed by adding $NaHCO_3$ (152 mg, 1.9 equiv). To the mixture was added a solution of Fmoc-OSu (374 mg, 1.2 equiv) in dioxane (1 mL) over 15 min. The solution was stirred for 24 h at room temperature. All the organic solvent was removed and dissolved in 5 mL of water. 1M of HCl was added to adjust pH to be 3 to 4. The aqueous was extracted with ethyl acetate. The combined organic solution was dried over Na_2SO_4. The organic solution was concentrated and the white solid (415 mg, 99% yield) was obtained by flash column chromatography.^{1}H NMR (400 MHz, d_6-DMSO):7.90 (d, *J* = 7.60 Hz, 2H), 7.71 (d, *J* = 7.30 Hz, 2H), 7.60 (d, *J* = 8.0 Hz, 1H), 7.42 (t, *J* = 7.45 Hz, 2H), 7.33 (t, *J* = 7.34 Hz, 2H), 4.33-4.28 (m, 2H), 4.25-4.20 (m, 1H), 3.93-3.86 (m, 1H), 1.62-1.52 (m, 1H), 1.51-1.42 (m, 1H), 1.42-1.35 (m, 2H), 0.99 (s, 3H) ppm;

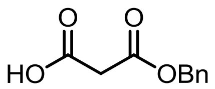

3-(benzyloxy)-3-oxopropanoic acid[5]To a solution of malonic acid (6.55 g, 62.94 mmol) in acetonitrile (100 mL) was added Et_3N (5.6 mL 1.0 equiv) and BnBr (5 mL, 1.0 equiv), respectively. The solution was stirred for 3 h at reflux temperature. Then the reaction flask was cooled to 0°C and 1 M HCl was added to adjust the pH to be 3 to 4. The aqueous solution was extracted with ethyl acetate. The organic layer was pooled and evaporated to dry. The oil was re-dissolved in sat. $NaHCO_3$. The aqueous solution was washed with diethyl ether to remove the di-benzyl malonate. At 0 ^{o}C 1 M HCl was added to the aqueous solution to adjust the pH to be 3 to 4. The suspension was extracted with ethyl acetate. The combined organic layer was dried over

Na_2SO_4. The organic solution was concentrated and the white solid (6.7 g, 82% yield) was obtained without any other purification.^{1}H NMR (400 MHz, $CDCl_3$): 7.37 (brs, 5H), 5.22 (s, 2H), 3.49 (s, 2H) ppm;

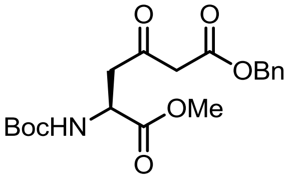

(S)-6-benzyl 1-methyl 2-((*tert*-butoxycarbonyl)amino)-4-oxohexanedioate (ii)[6,7]A: To a solution of Boc-Asp(OH)-OMe (2.0 g, 8.09 mmol) in anhydrous THF was added 1,1'-Carbonyldiimidazole (1.44 g, 1.1 equiv). Then the solution was stirred for 3.5 h at room temperature. B: To a solution of monobenzyl malonate (1.73 g, 1.1 equiv) in anhydrous THF was added isopropyl magnesium chloride (8.9 mL, 2M in THF, 2.2 equiv) over 20 min at 0 °C under the protection of argon. The solution was stirred at 0 °C for 30 min, and then was allowed to stir at 50 °C for 30 min. At 0 °Csolution A was added into solution B via syringe. The mixture was stirred at room temperature overnight. 0.1 M HCl was added to quench the reaction and the mixture was extracted with ethyl acetate. The combined organic layer was washed with brine and dried over Na_2SO_4. The organic solution was concentrated and the white solid (2.62 g, 85% yield) was obtained by flash column chromatography. ^{1}H NMR (400 MHz, $CDCl_3$):7.36 (brs, 5H), 5.46 (s, 1H), 5.17 (s, 2H), 4.53 (s, 1H), 3.70 (s, 3H), 3.51 (s, 2H), 3.28-3.06 (m, 2H), 1.44 (s, 9H) ppm;

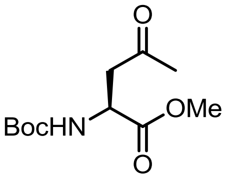

(S)-methyl 2-((*tert*-butoxycarbonyl)amino)-4-oxopentanoate (iii)[7]To a solution of starting material (2.62 g, 6.91 mmol) in methanol was added Pd/C (10% Pd on carbon, 262 mg, 10 mol%). The suspension was stirred for 4 h with a H_2 balloon as the hydrogen source. The suspension was filtered through Celite® to remove the Pd/C. And the filtrate was concentrated and the product (oil, 1.4 g, 83% yield) was obtained by flash column chromatography. ^{1}H NMR (400 MHz, $CDCl_3$): 5.48 (d, J = 7.40 Hz, 1H), 4.50-4.48 (m, 1H), 3.73 (s, 3H), 3.22-3.16 (m, 1H), 2.99-2.93 (m, 1H), 2.17 (s, 3H), 1.45 (s, 9H) ppm;

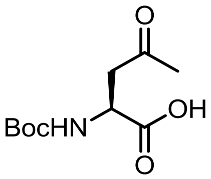

(S)-2-((*tert*-butoxycarbonyl)amino)-4-oxopentanoic acid (iv)[8]To a solution of ester (1.4 g, 5.71 mmol) in THF (14 mL) was added 1 M LiOH (273 mg, 2.0 equiv) over 20 min. After stirred at room temperature for 1 h, the solution was cooled to 0 °C and 0.1 M HCl was added to adjust pH to be 3 to 4. The suspension was extracted with ethyl acetate. The combined organic layer was washed with brine and dried over Na_2SO_4. The white solid (1.3 g, 98% yield) was obtained by evaporating all the solvent and used for next step without further purification. ^{1}H NMR (400 MHz,

$CDCl_3$): 5.56 (d, *J* = 8.40 Hz, 1H), 4.54-4.52 (m, 1H), 3.19-2.94 (m, 2H), 2.19 (s, 3H), 1.44 (s, 9H) ppm;

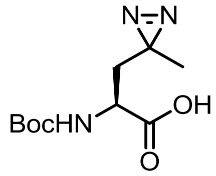

(S)-2-((*tert*-butoxycarbonyl)amino)-3-(3-methyl-3H-diazirin-3-yl)propanoic acid (v)[8] Ketone (1 g, 4.32 mmol) was dissolved in about 20 mL of liquid ammonia by ice-acetone condenser. The solution was stirred for 5 h at –30 °Cto –40 °C. Then the reaction flask was cooled to –50 °C and a solution of hydroxylamine-*O*-sulfonic acid (0.587 g, 1.2 equiv) in anhydrous methanol was dropped into the mixture over 30 min. The reaction was stirred at –30°C to –40 °C for another 10 h and then allowed to room temperature. Then ammonia was evaporated and the suspension was filtered through Celite® to remove the solid. The filter cake was washed with several portions of anhydrous methanol. The combined washings were concentrated until no smell of ammonia could be detected. The oil was re-dissolved in 10 mL of anhydrous methanol and cooled to 0 °C. TEA (1.21 mL, 2.0 equiv) was added followed by the addition iodine solution in MeOH until a brown color persisted. The reaction was stirred at room temperature for 1h, then concentrated and re-dissolved in ethyl acetate. The organic solution was washed by water and brine, respectively; dried over Na_2SO_4. The organic solution was concentrated and the colorless liquid (0.32 g, 54% yield) was obtained by flash column chromatography. ^{1}H NMR (400 MHz, MeOD): 4.07-4.03 (m, 1H), 1.95-1.91 (m, 1H), 1.62-1.56 (m, 1H), 1.44 (s, 9H), 1.05 (s, 3H) ppm;

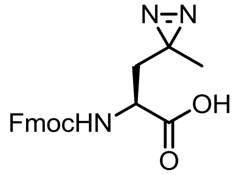

(S)-2-((((9H-fluoren-9-yl)methoxy)carbonyl)amino)-3-(3-methyl-3H-diazirin-3-yl)propanoic acid (vii)[8] The Boc-protected diazirine (0.3 g, 1.23 mmol) was dissolved in TFA/DCM (1:1, 10 mL). The mixture was stirred at room temperature for 1 h. Then the solvent was removed and the residue was redissolved in water (5 mL) and dioxane (10 mL) followed by adding $NaHCO_3$ (197 mg, 1.9 equiv). To the mixture was added a solution of Fmoc-OSu (499 mg, 1.2 equiv) in dioxane (1 mL) over 15 min. The solution was stirred at room temperature for 24 h. All the organic solvents were removed and dissolved in 5 mL of water. 1M HCl was added to adjust pH to be 3 to 4. The aqueous was extracted with ethyl acetate. The combined organic solution was dried over Na_2SO_4. The organic solution was concentrated and the white solid (0.44 g, 97% yield) was obtained by flash column chromatography. ^{1}H NMR (300 MHz, MeOD) δ 7.79 (d, *J* = 7.30 Hz, 2H), 7.71 (d, *J* = 7.30 Hz, 2H), 7.38 (t, *J* = 7.30 Hz, 2H), 7.30 (t, *J* = 7.10 Hz, 2H), 4.44 – 4.29 (m, 2H), 4.25 (t, *J* = 6.80 Hz, 1H), 4.12 (dd, *J* = 10.60, 4.30 Hz, 1H), 2.00 (dd, *J* = 15.00, 4.40 Hz, 1H), 1.64 (dd, *J* = 14.90, 10.60 Hz, 1H), 1.05 (s, 3H) ppm;

Synthesis of Rhodamine-conjugated BSA

Scheme 2. Synthesis of Rhodamine-conjugated BSA

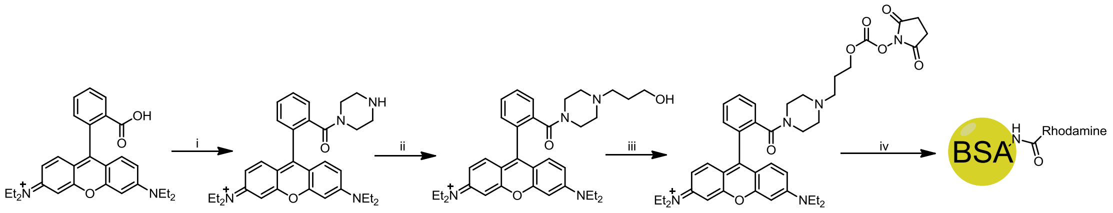

Conditions: (i) (a) 1 M of NaOH, extraction; (b) $AlMe_3$, piperazine, DCM, 63% yield; (ii) 3-bromopropanol, DIPEA, DMF, 56% yield; (iii) *N*,*N'*-disuccinimidyl carbonate, pyridine, 42% yield; (iv) BSA, PBS buffer, pH 8.3.

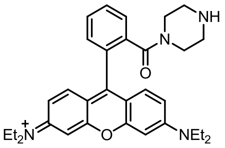

Rhodamine B piperazine amide (Rh-1)[9]. Rhodamine B (3.3 g, 6.9 mmol) was dissolved in ethyl acetate. The solution was washed by 1M NaOH. The aqueous layer was extracted with ethyl acetate. The combined organic solution was washed with brine and dried over Na_2SO_4. The intermediate was obtained by evaporating all the solvent. And it was used for the next step without any further purification. To a solution of piperazine (4.0 equiv) in anhydrous DCM was slowly dropped trimethyl aluminum (2.0 M in toluene, 2.0 equiv) at room temperature. The white precipitate was observed after 1 h stirring. To this suspension was added a solution of prepared Rhodamine B intermediate in anhydrous DCM carefully. After stirring at reflux temperature 24 h, HCl (0.1 M) was added to quench the reaction. The heterogeneous solution was filtered to remove the precipitate. The solution was extracted with DCM. The combined extracts were dried over Na_2SO_4. The solution was concentrated and the product was obtained (2.2 g, 63% yield) with flash column chromatography.^{1}H NMR (400 MHz, $CDCl_3$) δ 10.19 (s, 1H), 7.74-7.62 (m, 2H), 7.59 (d, *J* = 7.10 Hz, 1H), 7.33 (d, *J* = 7.00 Hz, 1H), 7.19 (d, *J* = 9.40 Hz, 2H), 6.99 (d, *J* = 8.10 Hz, 2H), 6.73 (s, 2H), 3.89 (d, *J* = 20.50 Hz, 4H), 3.70 (s, 4H), 3.58 (dd, *J* = 14.60, 7.20 Hz, 4H), 3.11 (d, *J* = 24.50 Hz, 4H), 1.34 (t, *J* = 7.00 Hz, 12H) ppm;

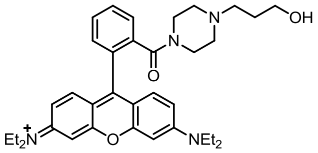

Rhodamine B 4-(3-hydroxypropyl)piperazine amide (Rh-2)[9] To a solution of Rh-1(87 mg, 0.17 mmol) in anhydrous DMF (0.5 mL) was added DIPEA (53 μL, 1.8 equiv) and 3-bromopropanol (20 μL, 1.3 equiv), respectively. The reaction was stirred at room temperature overnight. Then another portion of DIPEA (1.8 equiv) and 3-bromopropanol (1.3 equiv) was added and the reaction was kept stirring for another 2 h. All the solvents were removed and the product (54 mg, 56% yield) was purified by flash column chromatography.^{1}H NMR (400 MHz, MeOD):7.78-7.76

(m, 2H),7.68-7.66 (m, 1 H), 7.54-7.51 (m, 1H), 7.28 (d, *J* = 9.60 Hz, 2H), 7.09 (dd, *J* = 9.60, 2.40 Hz, 2H), 6.99 (d, *J* = 2.40 Hz, 2H), 3.70 (q, *J* = 7.20 Hz, 8H), 3.57 (t, *J* = 6.00 Hz, 2H), 3.42 (br, 4H), 2.42 (t, *J* = 7.20 Hz, 2H), 2.30 (br, 4H), 1.70-1.63 (m, 2H), 1.32 (t, *J* = 7.20 Hz, 12H) ppm;

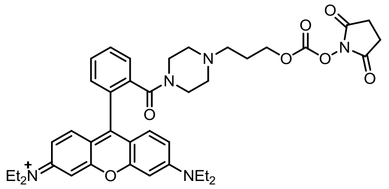

Rhodamine B 4-(3-N-hydroxysuccinimidyloxocarbonyl)-propyl)piperazine amide (Rh-3)[9](liquid, 42%) To a solution of Rh-2 (50 mg, 0.082 mmol) in anhydrous pyridine (0.5 mL) was added *N*,*N*'-disuccinimidyl carbonate (DSC) (63 mg, 3.0 equiv). The solution was stirred at room temperature for 2 h. Then another portion of DSC (63 mg, 3.0 equiv) was added into the reaction flask and the reaction was kept stirring for another 2 h. Then the reaction was quenched by adding water. All the solvent was removed and the product was purified by flash column chromatography with 8% MeOH in $CHCl_3$. ^{1}H NMR (300 MHz, MeOD) δ7.78 (dd, *J* = 5.20, 3.70 Hz, 2H), 7.73-7.62 (m, 1H), 7.54 (dd, *J* = 5.80, 2.90 Hz, 1H), 7.30 (d, *J* = 9.50 Hz, 2H), 7.10 (dd, *J* = 9.50, 2.30 Hz, 2H), 7.00 (d, *J* = 2.30 Hz, 2H), 4.39 (t, *J* = 6.00 Hz, 1H), 4.14 (t, *J* = 6.30 Hz, 1H), 3.88-3.55 (m, 8H), 3.42 (s, 4H), 2.83 (d, 2H), 2.70 (s, 2H), 2.53-2.31 (m, 2H), 2.23 (s, 4H), 1.98-1.67 (m, 2H), 1.34 (t, *J* = 7.00 Hz, 12H) ppm;

The preparation of rhodamine-conjugated BSA and the determination of degree of labeling

10 mg bovine serum albumin (BSA) was dissolved in 1 mL of 0.1 M sodium bicarbonate buffer (pH 8.3).To this mixture was slowly added a solution of Rhodamine-OSu in DMSO (50–100 μL, 10 mg/mL). Then the mixture was incubated for 1 h at room temperature with continuous stirring. After 1 h, 0.1 mL of freshly prepared 1.5 M hydroxylamine (pH 8.5) was added to quench the reaction. The solution was incubated for 1 h at room temperature. Then the samplewas dialyzed for 3 times with 1XPhosphate Buffered Saline (PBS buffer)(pH = 7.4). The absorbance of the protein-dye conjugated at 280 nm (A_{280}) and at λ_{max} for the dye (A_{max}) were measured. Then the protein concentration was determined to be 1.0 mg/mL according to the following formula:

$$A_{protein} = A_{280} - A_{max}(\mathrm{CF})$$

$$\mathrm{CF} = \frac{A_{280\,free\ dye}}{A_{\max free\ dye}}$$

Finally, the degree of labeling (D.O.L.) was determined to be 0.48 according the following formula:

$$\mathrm{DOL} = \frac{A_{max} \times MW}{[protein]\varepsilon_{dye}}$$

Where *MW*= the molecular weight of the protein,ε_{dye} = the extinction coefficient of the dye at its absorbance maximum, and the protein concentration is in mg/mL.

Reference:

(1) J. Du, Y. Zhou, X. Su, J. J. Yu, S. Khan, H. Jiang, J. Kim, J. Woo, J. H. Kim, B. H. Choi, B. He, W. Chen, S. Zhang, R. A. Cerione, J. Auwerx, Q. Hao and H. Lin, *Science*, 2011, 334,

806-809.
(2) B. P. Hubbard, A. P. Gomes, H. Dai, J. Li, A. W. Case, T. Considine, T. V. Riera, J. E. Lee, S. Y. E, D. W. Lamming, B. L. Pentelute, E. R. Schuman, L. A. Stevens, A. J. Ling, S. M. Armour, S. Michan, H. Zhao, Y. Jiang, S. M. Sweitzer, C. A. Blum, J. S. Disch, P. Y. Ng, K. T. Howitz, A. P. Rolo, Y. Hamuro, J. Moss, R. B. Perni, J. L. Ellis, G. P. Vlasuk and D. A. Sinclair, *Science*, 2013, 339, 1216-1219.
(3) S. S. More and R. Vince, *J. Med. Chem.*, 2009, 52, 4650-4656.
(4) M. R. P. Miquel Vila-Perelló, Frej Tulin, and Tom W. Muir, *J. Am. Chem. Soc.*, 2007, 129, 8068-8069.
(5) S. A. L. Thetiot-Laurent, B. Nadal and T. Le Gall, *Synthesis*, 2010, 2010, 1697-1701.
(6) G. F. Kaufmann, R. Sartorio, S.-H. Lee, J. M. Mee, L. J. Altobell, D. P. Kujawa, E. Jeffries, B. Clapham, M. M. Meijler and K. D. Janda, *J. Am. Chem. Soc.*, 2006, 128, 2802-2803.
(7) A. R. Mohite and R. G. Bhat, *J. Org. Chem.*, 2012, 77, 5423-5428
(8) A. L. MacKinnon, J. L. Garrison, R. S. Hegde and J. Taunton, *J. Am. Chem. Soc.*, 2007, 129, 14560-14561.
(9) T. Nguyen and M. B. Francis, *Org. Lett.*, 2003, 5, 3245-3248.
